# The Epstein Barr virus circRNAome

**DOI:** 10.1371/journal.ppat.1007206

**Published:** 2018-08-06

**Authors:** Nathan Ungerleider, Monica Concha, Zhen Lin, Claire Roberts, Xia Wang, Subing Cao, Melody Baddoo, Walter N. Moss, Yi Yu, Michael Seddon, Terri Lehman, Scott Tibbetts, Rolf Renne, Yan Dong, Erik K. Flemington

**Affiliations:** 1 Department of Pathology, Tulane University School of Medicine, Tulane Cancer Center, New Orleans, LA, United States of America; 2 Department of Structural and Cellular Biology, Tulane University School of Medicine, Tulane Cancer Center, New Orleans, LA, United States of America; 3 Roy J. Carver Department of Biochemistry, Biophysics and Molecular Biology, Iowa State University, Ames, IA, United States of America; 4 Reprocell USA, Beltsville, MD, United States of America; 5 Department of Biochemistry and Molecular Biology, University of Florida College of Medicine, Gainesville, FL, United States of America; 6 Department of Molecular Genetics and Microbiology, University of Florida, Gainesville, FL, United States of America; Duke University Medical Center, UNITED STATES

## Abstract

Our appreciation for the extent of Epstein Barr virus (EBV) transcriptome complexity continues to grow through findings of EBV encoded microRNAs, new long non-coding RNAs as well as the more recent discovery of over a hundred new polyadenylated lytic transcripts. Here we report an additional layer to the EBV transcriptome through the identification of a repertoire of latent and lytic viral circular RNAs. Utilizing RNase R-sequencing with cell models representing latency types I, II, and III, we identified EBV encoded circular RNAs expressed from the latency Cp promoter involving backsplicing from the W1 and W2 exons to the C1 exon, from the EBNA BamHI U fragment exon, and from the latency long non-coding RPMS1 locus. In addition, we identified circular RNAs expressed during reactivation including backsplicing from exon 8 to exon 2 of the LMP2 gene and a highly expressed circular RNA derived from intra-exonic backsplicing within the BHLF1 gene. While expression of most of these circular RNAs was found to depend on the EBV transcriptional program utilized and the transcription levels of the associated loci, expression of LMP2 exon 8 to exon 2 circular RNA was found to be cell model specific. Altogether we identified over 30 unique EBV circRNAs candidates and we validated and determined the structural features, expression profiles and nuclear/cytoplasmic distributions of several predominant and notable viral circRNAs. Further, we show that two of the EBV circular RNAs derived from the RPMS1 locus are detected in EBV positive clinical stomach cancer specimens. This study increases the known EBV latency and lytic transcriptome repertoires to include viral circular RNAs and it provides an essential foundation and resource for investigations into the functions and roles of this new class of EBV transcripts in EBV biology and diseases.

## Author summary

Our understanding of the extent of EBV transcriptome complexity has recently come to light with discoveries of viral microRNAs, snoRNAs, and a diverse set of transcript isoforms expressed during reactivation. While EBV utilizes distinct transcriptional programs during it’s normal infection cycle, the latency programs are critical drivers of EBV associated oncogenesis where they activate oncogenic pathways without killing the cell *via* lytic viral replication. Further, pathogenic latency programs typically express minimal levels of viral protein coding genes and instead rely on non-coding RNA mechanisms to alter the host cell environment. The utilization of non-coding RNAs in these settings is presumably an effort to minimize infected cell killing by the immune system through adaptive immune responses. Using tissue culture models representing the spectrum of EBV transcriptional states that occur in vivo, we report here that EBV expresses the circular class of predominantly non-coding RNAs. This stands to reveal a previously unknown set of players in EBV associated biology and disease. The findings reported here set a key platform from which future functional and potentially clinical related studies can be carried out to bring the initial findings from discovery to a new understanding of EBV biology and disease.

## Introduction

Epstein Barr virus (EBV) is a human oncogenic gamma herpesvirus that is carried by greater than 90% of the world’s population. While infection with EBV is generally asymptomatic, the virus persists for the lifetime of the host through an intricate and dynamic interplay with the host immune system, achieved in part through the virus’ utilization of multiple distinct gene expression programs. Initial infection through salivary exchange results in infection of the oral epithelium where the full repertoire of viral “lytic” genes is expressed to facilitate local amplification of virus titers. The virus is then transmitted to circulating naïve B-cells where a “latency type III” viral gene expression program is utilized (Latent Membrane Proteins (LMPs) -1 and -2, Epstein Barr Nuclear Antigens (EBNAs) -1, -2, -3A, -3B, -3C, and -LP and the non-coding transcripts, EBER1, EBER2, v-sisRNAs, v-snoRNA, RPMS1, and a set of viral miRNAs encoded within the introns of RPMS1) [1–4]. The expression of this full repertoire of latency genes facilitates potent B-cell activation and proliferation, a unique mechanism to expand the infected B-cell population in the host (i.e. independently of new virus production and *de novo* infection). Once an adaptive immune response is mounted to the antigenic latency proteins, the virus adapts through transitioning to (and/or selection for) more restricted latency gene expression programs, type II latency (EBER1, EBER2, RPMS1, viral miRNAs, EBNA1 and LMP1 and LMP2), type I latency (EBER1, EBER2, RPMS1, viral miRNAs and EBNA1) or type 0 latency (only non-coding RNAs, EBER1, EBER2, RPMS1, and viral miRNAs) [1, 2] where the virus persists at low levels with little detriment to the host. In the context of systemic immune suppression (e.g. HIV infection or clinically induced), variable expression of growth promoting type III latency genes can be better tolerated, which often leads to the development of B-cell lymphomas. In immune-competent individuals the viral utilization of non-coding RNAs in addition to low level type I or type II protein coding latency gene expression, perhaps tolerated through local tumor-immune suppression, provides one or more “hits” towards oncogenic progression with minimal impact from the immune system.

The role of EBV latency proteins in contributing to the oncogenic phenotype has been the topic of study for many years. More recent studies have demonstrated the importance of the EBV non-coding RNAs, RPMS1, EBV miRNAs, and EBER1/2 (expressed across all EBV infected tumor types) in contributing to the tumor phenotype through promoting cell cycle progression, inhibiting innate and adaptive immune responses, blocking apoptosis, etc [5–12]. Utilizing non-coding RNAs to modulate host cell signaling pathways is likely an important viral strategy for molding the host cell environment in ways that support of the virus’ needs without eliciting immune clearance.

Circular RNAs [13–15] are another conserved class of predominantly non-coding RNAs that have gained increased attention in recent years [16–18]. circRNAs are produced by backsplicing of a 3’ splice donor to an upstream 5’ splice acceptor, generating a covalently closed RNA molecule with increased stability due to their lack of exonuclease susceptible 5’ or 3’ substrates. While our understanding of the roles, functions, and mechanisms of action of circRNAs remains nascent, many studies have pointed to non-coding functions such as acting as microRNA sponges (e.g. ciRS-7 [19]), through *cis* regulation of transcription [20], and through modulating RNA splicing [21]. In addition, however, a subset of circRNAs may be translated to form smaller peptide derivatives of the parental genes [22–24]. Given the conserved nature of circRNAs and findings that the majority of circRNAs likely play non-coding regulatory roles, we sought to determine whether EBV utilizes this class of transcripts to help facilitate regulation of host cell signaling without solicitating an adaptive immune response. Here we report a comprehensive analysis of the EBV circRNAome across latency types using a panel of cell lines modeling types I, II and III latency and in B-cell lymphomas and gastric cancers. Further, we report the lytic circRNAome using B-cell receptor cross-linking in two Burkitt’s lymphoma cell lines, Akata and Mutu I. These experiments identified an extensive repertoire of viral circRNAs that may play unique roles in different latency stages of EBV infection and in Burkitt’s and gastric cancers as well as those that may be important in viral lytic replication. Together, this work further expands the repertoire of the viral transcriptome to include the circular RNA class of RNAs and stands to set the stage for the discovery of new mechanisms through which EBV facilitates infection, persistence and oncogenesis.

## Results

### RNase R-sequencing for the detection of circular RNAs

As an initial exploration into whether EBV expresses circular RNAs, we analyzed our previously reported ribodepletion RNA-seq data from the EBV positive Burkitt’s lymphoma cell line, Akata, induced to undergo viral reactivation through B-cell receptor cross-linking (GEO series accession number, GSE52490) [25]. Using find_circ [26] to identify backsplice junction candidates, over a hundred unique backsplice junction calls were made across the EBV genome using this dataset (S1 Table and S1 Fig). Nevertheless, the bulk of these were low abundance and/or were deemed low-confidence upon realignment of raw RNA-seq data to conjoined sequences spanning a handful of these backsplice junctions and some backsplice junction calls mapped to simple repeat regions raising concerns about misalignment. Further, while for read alignments, the episomal EBV genome was linearized at a region displaying minimal transcription [25, 27], we were concerned that some backsplice calls could in fact represent forward splicing across the artificially linearized viral genome end-sequences.

**Fig 1:**
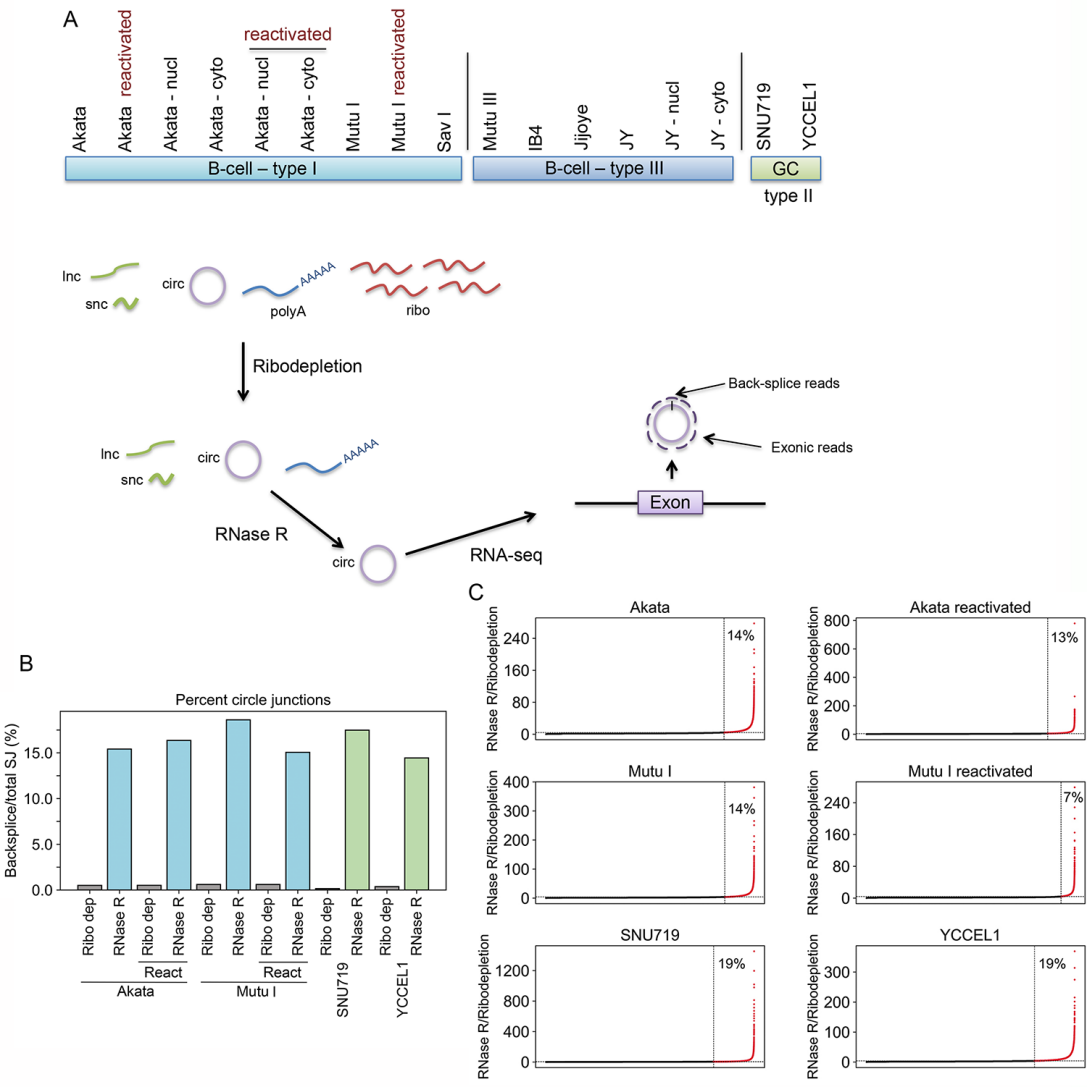
RNase R-sequencing across EBV latency types and reactivation. A) Cell models subjected to RNase R-seq and schematic of sequencing strategy. Nucl and cyto refer to nuclear and cytoplasmic fractions, respectively. B) Backsplice to total (backsplice plus forward splice) viral plus cellular read counts in ribodepletion-seq and RNase R-seq approaches. Note: similar backsplice to total SJ ratios were also observed in RNase R-seq analysis of all other cell lines/conditions shown in panel A. C) Scatter plots showing ratio of backsplice read counts (normalized to total spliced read counts) in RNase R vs ribodepletion sequencing for each junction detected in respective ribodepletion-seq datasets.

To decrease background and enhance the fidelity of circRNA detection, ribodepleted RNAs were treated with RNase R to digest linear RNAs prior to library preparation (Fig 1A). Decreasing the levels of linear RNAs through RNase R digestion also served to increase the circular to linear RNA ratios, thereby increasing the sensitivity of circRNA detection. This was considered important because while linear transcript detection is typically assessed by counting all reads mapping to the entire exonic regions, circRNAs can only be unequivocally assessed by the limited subset of reads spanning the backsplice junction.

To achieve a relatively comprehensive assessment of the EBV circRNAome, RNase R-sequencing was performed using cell models representing types I, II, and III latency transcription programs and during reactivation, and was performed using both lymphocyte and gastric cancer cell lines (Fig 1A). For six of these models we also performed ribodepletion-only RNA-seq analysis to help assess the relative performance of RNase R-seq. A global analysis of cellular plus viral reads showed that RNase R treatment resulted in a more than 20-fold higher ratio of backsplice to total splice junction reads, indicative of circular RNA enrichment in RNase R treated samples (Fig 1B). Also significant was the finding that less than 20% of backsplice junction calls from ribodepletion-seq data were enriched in RNase R treated samples by more than 4-fold (Fig 1C). This suggested that a substantial proportion of backsplice junction calls made using ribodepletion-seq data could be false positives. Consistently, of the 148 viral backsplice calls made from ribodepletion-seq data in reactivated Akata cells, only 17 were represented in the RNase R-seq data and 14 of these were enriched by more than 4-fold (S1 Table and S1 Fig). An additional eight unique EBV backsplice junctions with more than 5 reads were detected in the RNase R-seq data from reactivated Akata cells (S1 Table and S1 Fig) indicating higher sensitivity with RNase R-seq. Based on these analyses, we conclude that RNase R treatment prior to sequencing provides substantially improved specificity and sensitivity of circRNA detection over ribodepletion alone.

### Assessment of the EBV circRNAome

**Fig 2:**
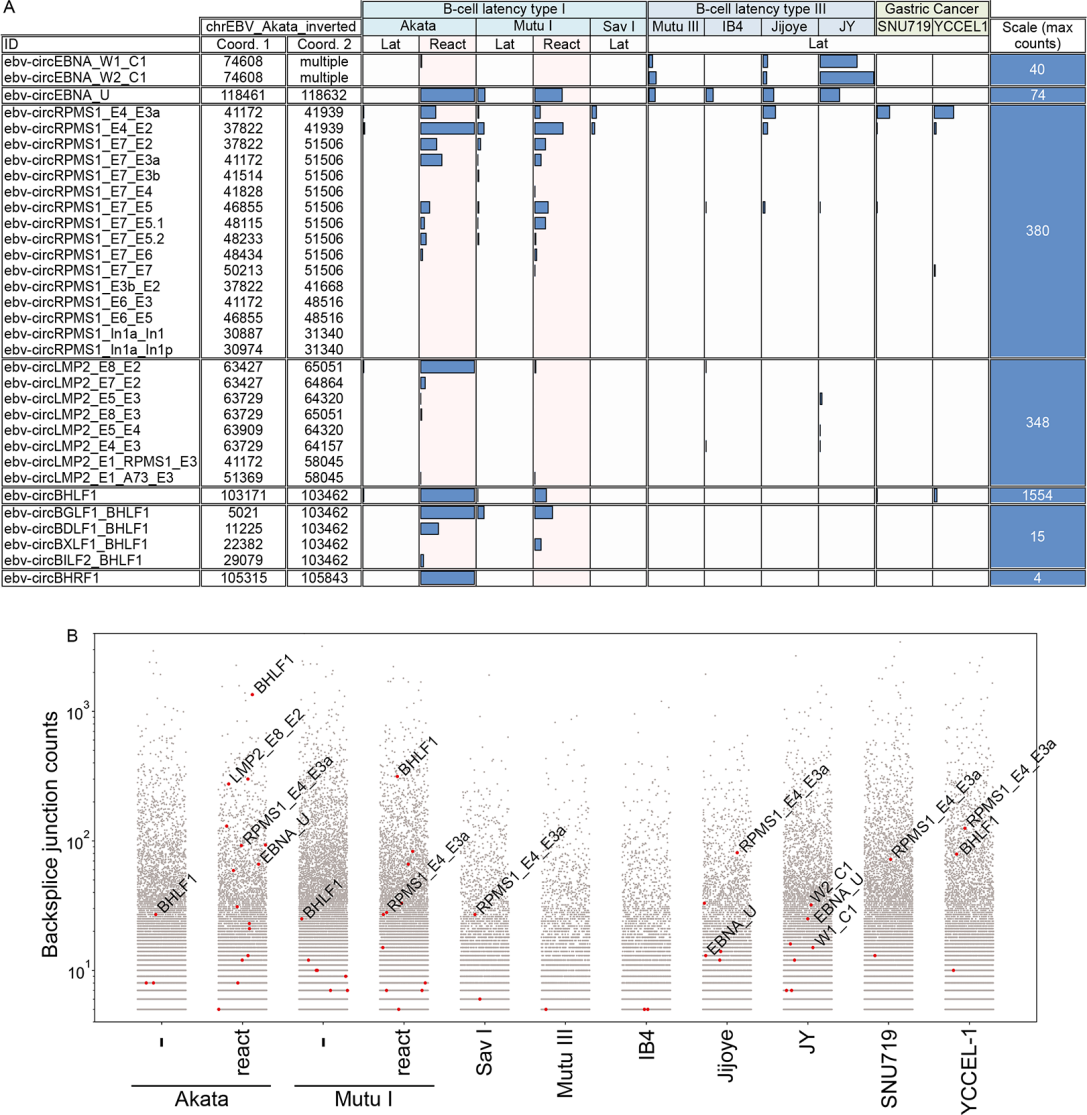
A) *In silico* validated EBV backsplice junctions. Bar graph displays junction spanning read counts (minimum 12 base overhang and 90% homology) derived from realignment to conjoined backsplice junctions. Note that some of the less abundant backsplices (e.g. several of the RPMS1 and the circBHRF1 backsplice junctions) met the 5 read minimum threshold in RNase R-seq of nuclear and/or cytoplasmic fractions but show less than 5 reads in any whole cell sequencing data displayed here. B) Scatter plot displaying viral backsplice read counts (red dots) in the context of cellular backsplice read counts (black dots) from find_circ analysis of RNase R-seq datasets.

Backsplice analysis across all RNase R-seq data sets shown in Fig 1A identified 35 unique EBV backsplice junctions supported by more than 5 reads in at least one sample. For each of these backsplice candidates, we visually assessed junction coverage following re-alignment of each dataset to conjoined sequences spanning each respective backsplice junction (S1 File). Two of these, RPMS1 exon 7 to exon 5_3 and RPMS1 intron 6 (see S1 File) likely met the minimum of 6 junction spanning read criteria due to PCR duplicates and were discarded from further analysis. Following this quality check filtering, 33 EBV backsplice junction candidates remained (Fig 2A).

While some of the EBV backsplice junctions showed relatively low coverage, others displayed reasonably robust representation (Fig 2A and S2 Table). To help gauge the significance of their expression levels, we plotted viral backsplice junction read counts across each dataset in the context of detected cellular backsplice junction read counts (Fig 2B) and we assessed their ranking among all cellular and viral backsplice sites detected (S3 Table). circEBNA_U and circRPMS1_E4_E3a, which were detected in both latency and reactivation conditions ranked 234^th^ and 34^th^ respectively in reactivated Akata cells (S3 Table). The less robustly expressed viral circRNAs, circEBNA_W1_C1 and circEBNA_W2_C1 were ranked 838^th^ and 1679^th^ in JY cells. Notably, although these backsplices were not detected in Mutu III, Jijoye, or reactivated Akata cells through our initial find_circ analysis, they were nevertheless detected in these cell lines following re-alignment to conjoined splice junctions (Fig 2A) and by RT-PCR (see below). Lastly, the lytic circRNA, circLMP2_E8_E2, was ranked 36^th^ in reactivated Akata cells and the lytic circBHLF1 was found to be the 2^nd^ most abundant circRNA in the nuclear/cytoplasmic fractionated reactivated Akata cells (S3 Table). This analysis demonstrated that many of the EBV circRNAs are expressed at levels that are comparable to cellular circRNAs (Fig 2B), raising the contention that they may have a comparable likelihood of functional significance.

### Circular RNA formation from the EBNA latency locus

**Fig 3:**
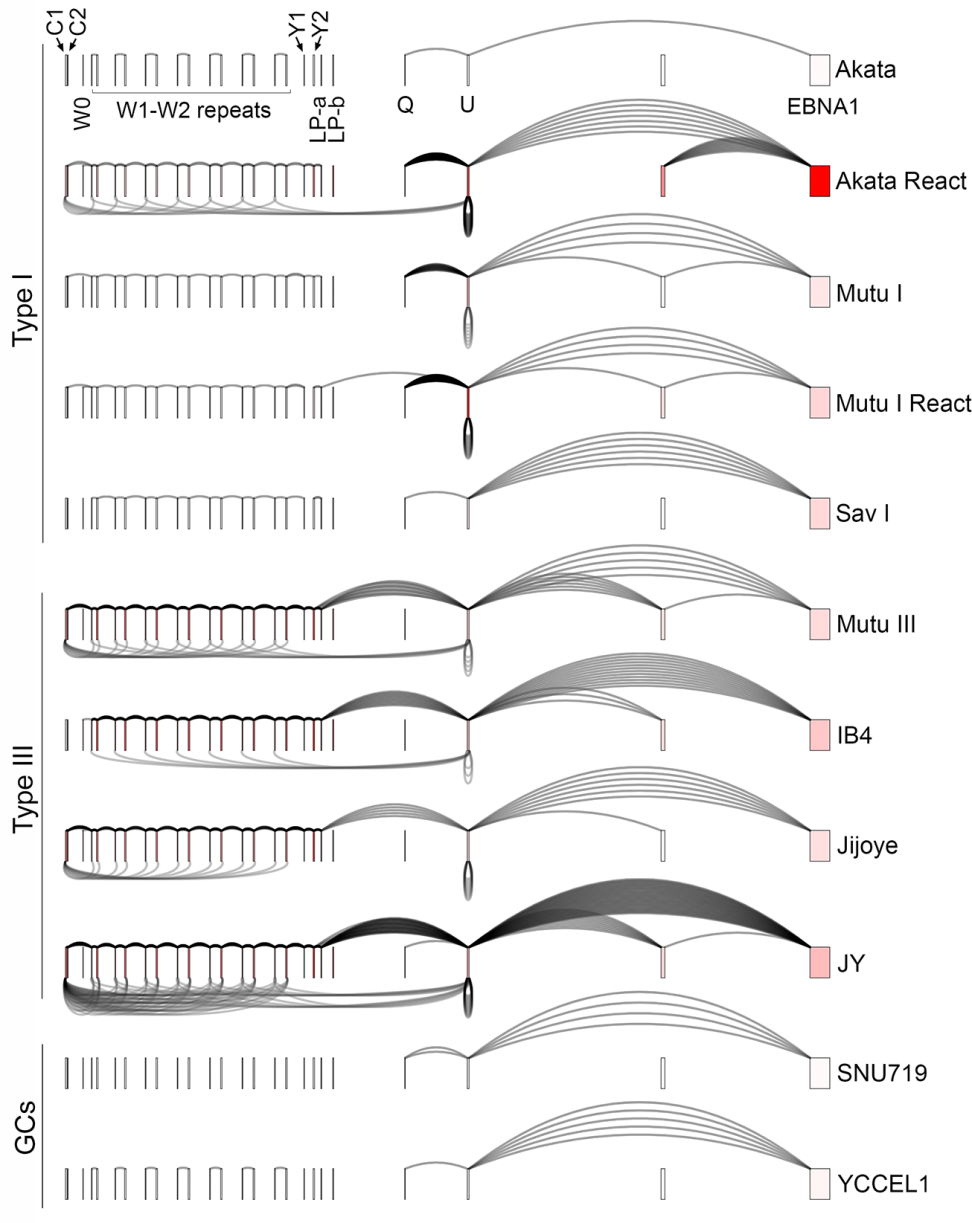
Graphical presentation of splicing and exon specific coverage of the EBNA latency locus. For context, backsplicing read counts (under arches) derived from RNase R-seq datasets are plotted with forward splicing (over arches) and coverage data (exon color intensity) derived from polyA-seq datasets. The number of arches (forward- and back-splicing) correspond to the number of junction spanning reads. Exon shading intensity reflects relative coverage levels across both exons and samples. Due to informatics issues associated with aligning to repeat sequences, we utilized a custom data processing method for analyzing the EBNA latency locus to more accurately assess coverage and splicing across the BamHI W repeat region.

In type III latency, expression of the EBV nuclear antigens, EBNA-LP, EBNA1, EBNA2, EBNA3A, EBNA3B and EBNA3C is derived through alternative splicing of transcripts originating from either the Bam HI C promoter (Cp; exon C1), or the Bam HI W promoter (Wp; exon W0) (Fig 3). Each of these transcripts contain multiple pairs of 5’ W1 and W2 exons derived from the Bam HI W repeats that are spliced to downstream exons containing different EBNA reading frames. Wp is primarily utilized during the initial stages of B-cell infection after which the virus switches to Cp utilization. Accordingly, most established type III latency cell lines show exclusive Cp usage (for example, Mutu III, Jijoye, and JY (Fig 3)) although some exceptions exist including the IB4 cell line (Fig 3). In type I and II latency where the only EBNA expressed is EBNA1, the C or W promoters are epigenetically silenced and EBNA1 is expressed from a third promoter, Qp, located downstream from the Bam HI W repeats (Fig 3).

Because of limitations associated with alignment to repeat sequences, we devised a custom data processing method for analyzing the EBNA latency locus to more accurately assess coverage and splicing across the BamHI W repeat region. Specifically, exon coverage and backsplicing read counts were obtained from alignments to a genome containing the EBNA latency locus with only a single Bam HI W repeat. For display, coverage and backsplice read counts associated with the W1 and W2 exons were divided by the appropriate number of exons/junctions to distribute these counts equivalently across 7 W repeats. A similar approach was used for forward splicing analyses except that two Bam HI W repeats were used for alignments to allow capturing of W2 to W1 splicing events.

**Fig 4:**
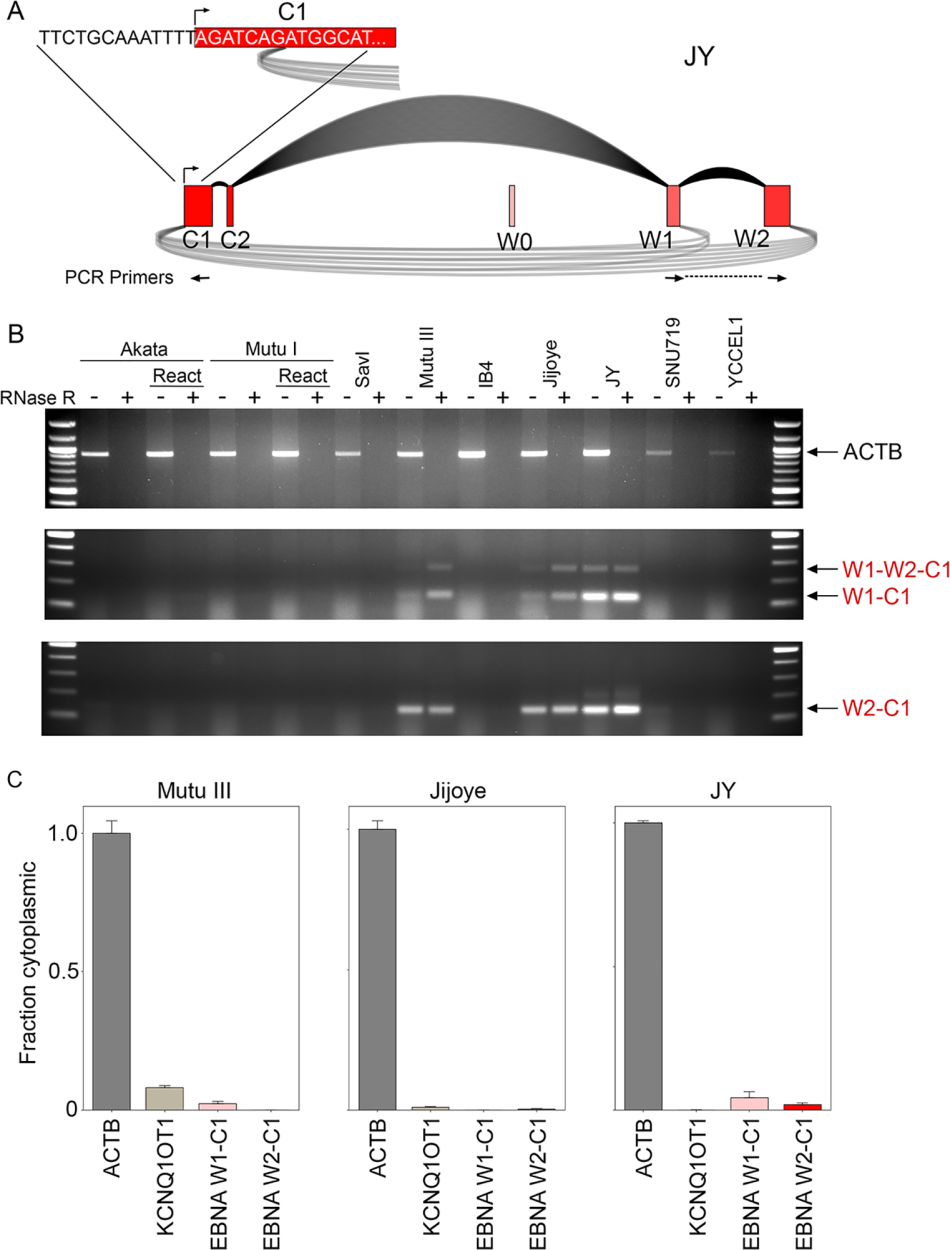
RT-PCR analysis of circEBNA_W1_C1 and circEBNA_W2_C1 circular RNAs. A) Detailed view of W1-to-C1 and W2-to-C1 backsplices and location of the novel C1 splice acceptor 8 bp downstream from the annotated Cp transcription initiation site. Positions of divergent W1 and C1 and W2 and C1 PCR primers are shown below respective exons. B) RNase R-resistance of circEBNA_W1_C1 and circEBNA_W2_C1 circular RNAs. Divergent C1 and W1 primers detect both W1-to-C1 backsplice junction and W1-to-W2-to-C1 splicing (middle panel–verified by sequencing). Divergent C1 and W2 primers detect the W2 to C1 backsplice junction (lower panel–verified by sequencing). These experiments were repeated with similar results. C) RT-qPCR of nuclear and cytoplasmic fractions using W1-to-C1 and W2-to-C1 divergent primers shows primarily nuclear localization for both circEBNA_W1_C1 and circEBNA_W2_C1 circular RNAs. Cytoplasmically localized ACTB and nuclear localized KCNQ1OT1 are shown for comparison. Fraction cytoplasmic is relative to ACTB (cytoplasmic) and the most nuclear localized junction in the respective cell lines. Error bars represent standard deviations and were derived from triplicate qPCR reactions. These experiments were repeated once with similar results. Method for calculating fraction cytoplasmic is outlined in the methods section.

As shown in Fig 3, backsplice reads spanning from the W1 and W2 exons to a novel splice acceptor in the C1 exon (Fig 4A) were detected in the Cp utilizing latency III cell lines, Mutu III, Jijoye, and JY but not in the Wp utilizing type III latency cell line IB4, the latency type I cell lines Akata, Mutu I and Sav I, or the latency type II cell lines, SNU719 and YCCEL1 (note; in Fig 3, RNase R-seq backsplice read counts are plotted with coverage and forward splice read count data from PolyA-seq data to provide a linear transcript expression context). W1 to C1 backsplicing was also observed in reactivated Akata cells, consistent with previous findings that Cp is induced during reactivation in Akata cells [25, 27]. Although not detected in our initial analysis, EBNA U to W1 exon backsplice reads were also observed in this analysis due to the informatics improvements made to accommodate BamHI W repeat alignment issues (Fig 3).

Using divergent primers in the W1 and C1 exons, both the W1-to-C1 backsplice junction and to a lesser extent, the W1-to-W2-to-C1 forward-and-backslice junctions were captured by RT-PCR (verified by sequencing) (Fig 4B). Both transcripts were resistant to RNase R digestion demonstrating the closed circular nature of both the W1-to-C1 and the W2-to-C1 containing RNAs (Fig 4B). Using divergent W2 and C1 primers, only a single band was identified that corresponded to the W2-to-C1 backsplice (verified by sequencing) and the associated RNA was resistant to RNase R digestion (Fig 4B). This data validates the expression of both circEBNA_W1_C1 and circEBNA_W2_C1 in type III latency and demonstrates the closed circular nature of both of these transcripts.

To assess subcellular localization, W1-to-C1 and W2-to-C1 divergent primer pairs were used to assess nuclear and cytoplasmic fractionated RNAs from Mutu III, Jijoye, and JY cells. This analysis indicated that both circEBNA_W1_C1 and circEBNA_W2_C1 are substantially enriched in the nuclear fractions (Fig 4C) (note: with the predominance of W1-C1 amplification in W1-to-C1 PCR reactions, the W1-to-C1 PCR products likely crossed the amplification threshold cutoff first; Ct values thereby most likely represent W1-to-C1 backsplicing). This nuclear localization is consistent with findings that circEBNA_W1_C1 and circEBNA_W2_C1 have little coding capacity. Strikingly, the novel C1 backsplice acceptor for these circRNAs is located only 8 nucleotides downstream from the Cp transcription initiation site (Fig 4A), leaving minimal upstream RNA sequences to bind splicing factors. Backsplicing to this splice acceptor could occur from low level upstream initiated transcripts or possibly through some novel mechanism, such as through interactions between splice donor RNA/protein complexes with promoter engaged (or proximal) transcription machinery or DNA.

The EBNA U exon is contained within EBNA transcripts driven by all three latency EBNA promoters, Cp, Wp, and Qp. The EBNA U backsplice is detected in types I and III latency (Fig 3 and S2 Fig) with the number of backsplice reads roughly reflecting the level of U exon coverage (Fig 3). No backsplices were detected in the two stomach cancer cell lines, SNU719 and YCCEL1 although this may be due to the very low level of U exon coverage observed in this setting. A fourth promoter located just upstream from Qp, referred to as Fp, gives robust Q-to-U-to-downstream EBNA ORF expression/splicing during reactivation. Accordingly, U exon expression is high during reactivation and consistently, a high level of exon U backsplicing is observed (Figs 2A and 3).

### Complex backsplicing at the RPMS1 locus

**Fig 5:**
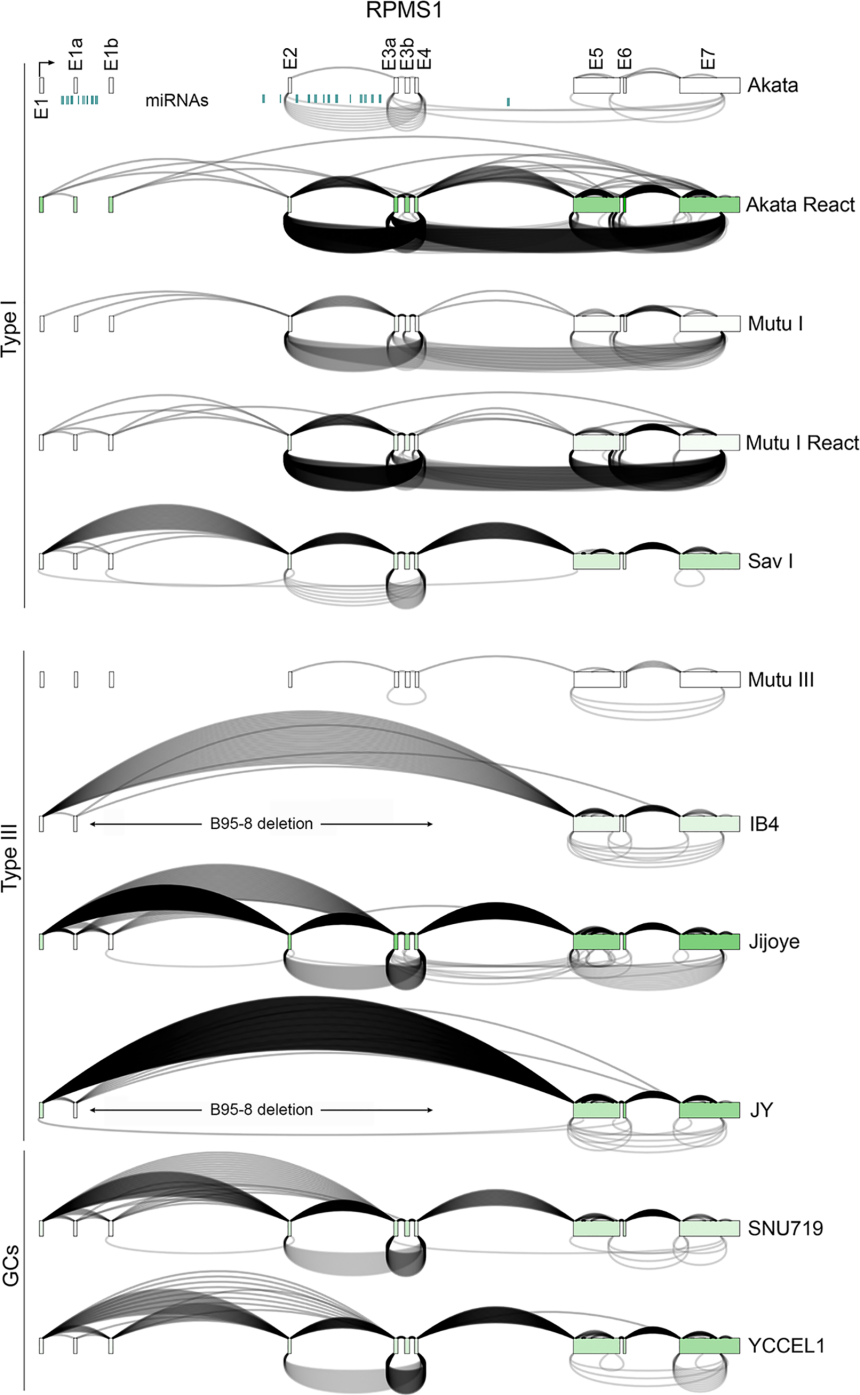
Graphical presentation of splicing and exon specific coverage of the RPMS1 latency locus. Backsplicing read counts (under arches) are derived from RNase R-seq datasets and forward splicing (over arches) and coverage data (exon color intensity) are derived from polyA-seq datasets. The number of arches (forward- and back-splicing) correspond to the number of junction spanning reads. Exon shading intensity reflects relative coverage levels across both exons and samples. The location of the B95-8 deletion in IB4 and JY cells is indicated.

**Fig 6:**
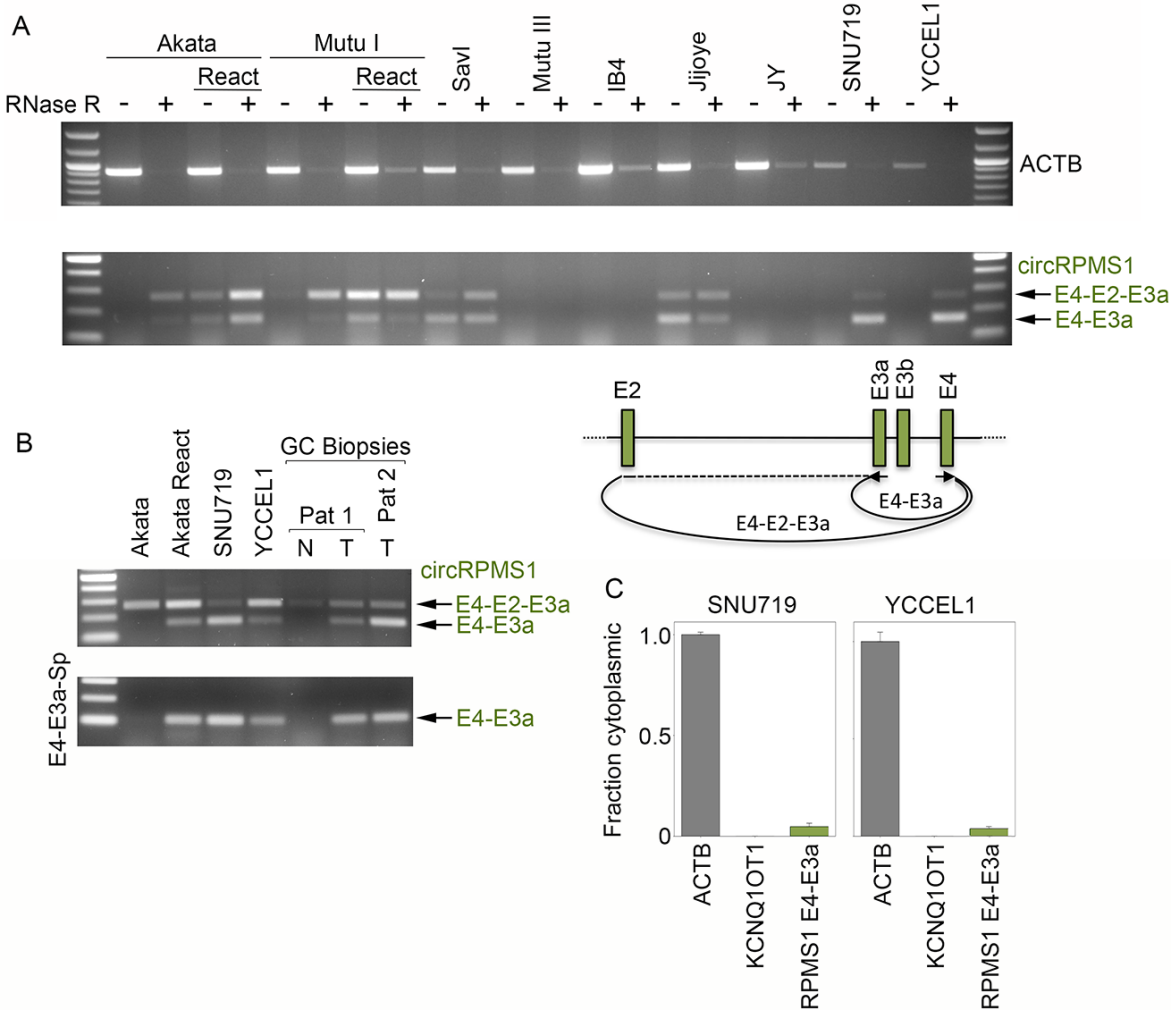
RT-PCR analysis of circRPMS1_E4_E3a and circRPMS1_E4_E2 circular RNAs. A) RNase R resistance of circRPMS1_E4_E3a and circRPMS1_E4_E2. Divergent primers within RPMS1 exons 4 and 3a detect both exon 4 to exon 3a and exon 4 to exon 2 (exon 4 to exon 2 to exon 3a) circular RNAs (validated by sequencing of excised fragments). All PCR reactions were repeated with similar results. B) circRPMS1_E4_E3a and circRPMS1_E4_E2 are detected in stomach cancer biopsies from two patients (Pat 1 and Pat 2). “N” refers to normal adjacent tissue and “T” refers to tumor tissue. All PCR reactions were repeated with similar results. C) RT-qPCR analysis using junction specific primers shows nuclear enrichment for circRPMS1_E4_E3a. Error bars represent standard deviations and were derived from triplicate qPCR reactions. These experiments were repeated once with similar results. Method for calculating fraction cytoplasmic is outlined in the methods section.

The latency RPMS1 locus is a complex transcriptional unit that gives rise to a set of alternatively spliced long non-coding RNAs and nearly 40 miRNAs that are derived from RPMS1 intronic sequences (Fig 5) [28–30]. Find_circ detected 16 unique backsplice junctions across the different tested conditions (Fig 2B) although the many of these were found to be of low abundance. During reactivation, a handful of RPMS1 backsplice junctions were found to be well represented including backsplices from a novel splice donor in exon 7 to exons 5, 3, and 2 and backsplices from exon 4 to exons 3a and 2 (Figs 2 and 5). Of these, circRPMS1_E4_E3a and circRPMS1_E4_E2 were found to be expressed across all latency types (note: both IB4 and JY contain the B95-8 deletion of exons 1b through 4 and accordingly, no circRPMS1_E4_E3a or circRPMS1_E4_E2 backsplice junctions were detected in these cell lines). Using a leftward primer in exon 3a and a rightward primer in exon 4, we detected both the exon 4 to exon 3a backsplice junction (validated by sequencing) and the exon 4 to 2 backsplice junction (exons 4-to-2-to-3a, validated by sequencing) (Fig 6A). Both backsplice junctions were detected across latency types although there was no detection in IB4 and JY due to the deletion of these sequences or in Mutu III cells which displays low expression of this locus. Further, both fragments were detected in RNase R treated RNAs, validating their closed circular nature (Fig 6A).

To determine whether the circRPMS1_E4_E3a and circRPMS1_E4_E2 circular RNAs could be detected in the patient tumor setting, RT-PCR was performed using RNAs from two EBV positive stomach cancer patient specimens that we used in previous studies [31, 32]. As shown in Fig 6B, both circRPMS1_E4_E3a and circRPMS1_E4_E2 were detected in each of these tumor samples demonstrating their expression in the natural patient tumor setting.

Analysis of nuclear/cytoplasmic distribution using exon 4 and exon 3a divergent primers in SNU719 and YCCEL1 cells where the circRPMS1_E4_E3a PCR signal predominates over the circRPMS1_E4_E2 signal (Fig 6A) revealed potential low level cytoplasmic localization but with predominant nuclear localization (Fig 6C). With the upstream splice acceptor for circRPMS1_E4_E3a flanking a microRNA cluster intron, circRPMS1_E4_E3a backsplicing could influence the regulation of microRNA processing, although such a scenario doesn’t preclude other possible nuclear functions of mature circRPMS1_E4_E3a RNA.

### Context specific expression of circular RNAs from the LMP2 locus

**Fig 7:**
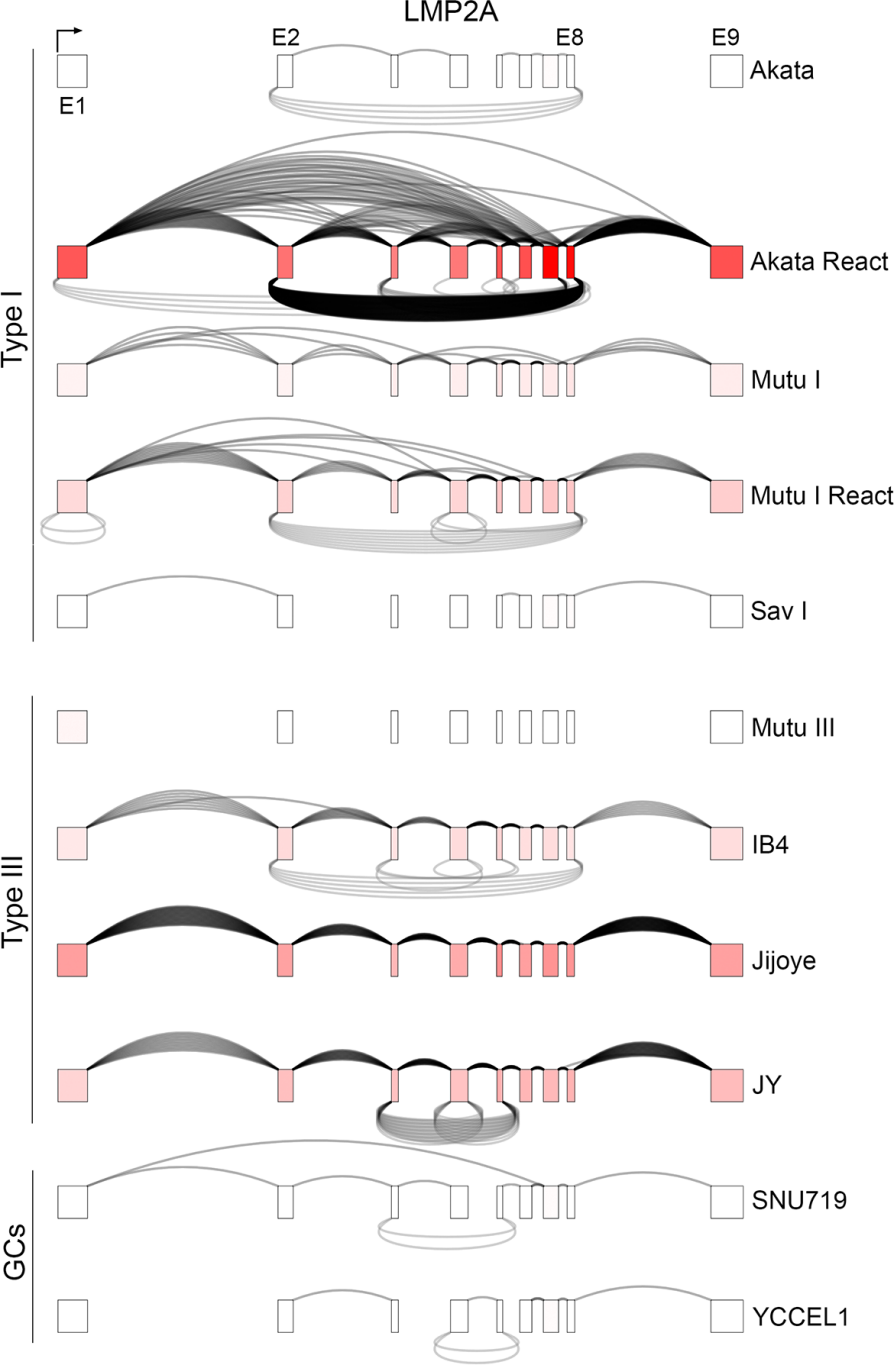
Graphical presentation of splicing and exon specific coverage of the LMP2 locus. Backsplicing read counts (under arches) are derived from RNase R-seq datasets and forward splicing (over arches) and coverage data (exon color intensity) are derived from polyA-seq datasets. The number of arches (forward- and back-splicing) correspond to the number of junction spanning reads. Exon shading intensity reflects relative coverage levels across both exons and samples.

While the LMP2 gene is typically expressed in type III latency cells but not in latency type I cells, reactivation in type I latency cells broadly activates the expression of latency genes, including LMP2A [33]. We have previously demonstrated that in contrast to the normal sequential forward splicing typically observed for LMP2A in type III latency, the LMP2 locus exhibits extensive alternative splicing during reactivation [25, 27, 34]. Adding to the complexity of this transcriptional unit during reactivation, we observe substantial backsplicing from exon 8 to exon 2 in reactivated Akata cells, with lower abundance backsplicing from exon 7 to exon 2 (Figs 7 and 2). While the extent of reactivation and expression of LMP2 in anti-IgM treated Mutu I cells is lower than that observed in reactivated Akata cells, LMP2 exon 8 to exon 2 backsplicing is also detected in this cell model (Figs 7 and 2). In contrast, the type III cell lines Jijoye and JY which constitutively express LMP2A show both a lack of alternative forward splicing and circLMP2_E8_E2 backsplicing suggesting context specific regulation of circLMP2_E8_E2 backsplicing (although some backsplicing from exons 5 to 3 and at exon 6 is observed in JY cells and exon 8 to 2 backsplicing is observed in IB4 cells) (Fig 7). This data indicates that circular RNA formation at the LMP2 locus is context dependent, raising the possibility that tissue specific splicing factors may control the levels of backsplicing through *cis* regulatory elements in the primary LMP2 transcript.

**Fig 8:**
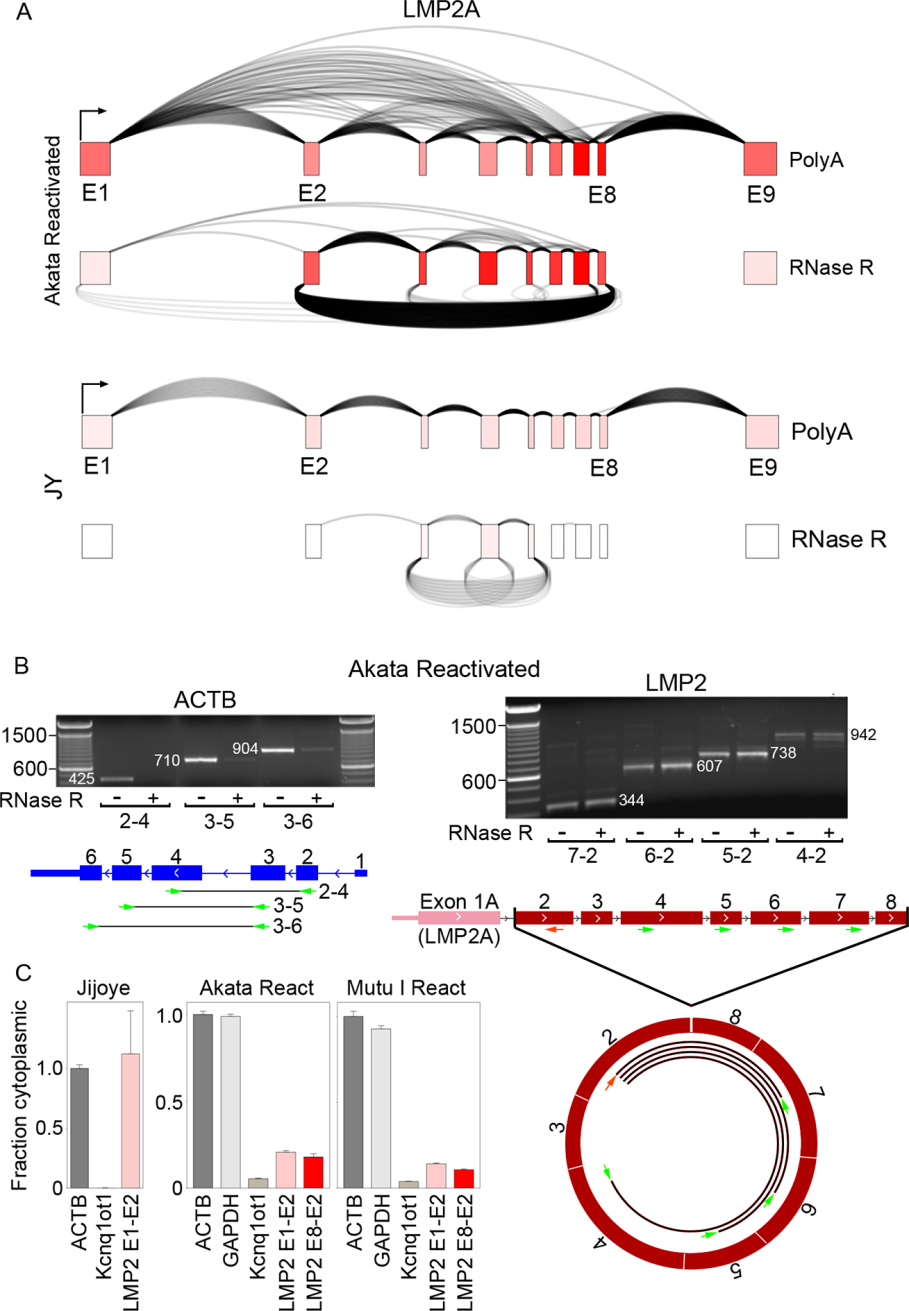
Exonic structure of circLMP2_E8_E2. A) Coverage and forward splicing enrichment of LMP2 exons 2 through 8 in RNase R-seq in reactivated Akata cells suggests exons 2 through 8 inclusion in circLMP2_E8_E2. Forward splicing (over arches), coverage (exon color intensity), and backsplicing (under arches) derived from polyA-seq and RNase R-seq in reactivated Akata cells and JY cells. B) RT-PCR using forward primers in exons 4 through 7 support a sequentially forward-spliced configuration of circLMP2_E8_E2. All PCR reactions were repeated with similar results. C) Nuclear/cytoplasmic distribution of circLMP2_E8_E2 using RT-qPCR. Cytoplasmically localized ACTB and nuclear localized KCNQ1OT1 are shown for comparison. Fraction cytoplasmic is relative to ACTB (cytoplasmic) and KCNQ1OT1 (nuclear). Error bars represent standard deviations and were derived from triplicate qPCR reactions. These experiments were repeated once with similar results. Method for calculating fraction cytoplasmic is outlined in the methods section.

The first exon of LMP2A encodes a cytoplasmic signaling domain and exons 2 through 8 encodes the multi-pass transmembrane domain, with exon 9 composed entirely of 3’ untranslated region (UTR) sequences [35]. A second form of LMP2, LMP2B, utilizes an alternative first exon located between LMP2A exon 1 and 2 [35]. With the first exon of LMP2B being composed entirely of 5’ UTR sequences, translation initiation of LMP2B occurs two bases into exon 2, giving rise to a dominant negative transmembrane inhibitor of LMP2A encoded by exons 2 through 8 [36]. Analysis of coverage and forward splicing data from RNase R-seq from reactivated Akata cells shows coverage and sequential forward splicing across each of exons 2 through 8 (with albeit low but detectable levels of alternative splicing) (Fig 8A) suggesting that the major circLMP2_E8_E2 species includes all coding sequences of LMP2B. To further explore the potential canonical forward spliced structure of circLMP2_E8_E2, we performed RT-PCR in reactivated Akata cells using a reverse primer in exon 2 and a panel of forward primers extending from each of exons 4–7. Each of these primer pairs gave rise to primary PCR fragments consistent with consecutive forward splicing across each of these exons (Fig 8B) which was confirmed by sequencing the excised bands. This indicates that the predominant structure of circLMP2_E8_E2 likely contains all of the coding sequences of the dominant negative LMP2B transcript. While the identification of possible function of circLMP2_E8_E2 will require further investigation, cellular distribution analysis shows low but significant cytoplasmic localization (Fig 8C), leaving open the possibility that this circular form of LMP2 could potentially represent a novel isoform of LMP2B. At the same time, analysis of previously published PAR-CLIP data from seven different EBV positive cell lines including lymphomas and the nasopharyngeal carcinoma cell line, C666 [37–40] did not reveal evidence of microRNA association with LMP2 exons 2–8 suggesting that circLMP2_E8_E2 may not play a microRNA sponge function.

### circBHLF1

**Fig 9:**
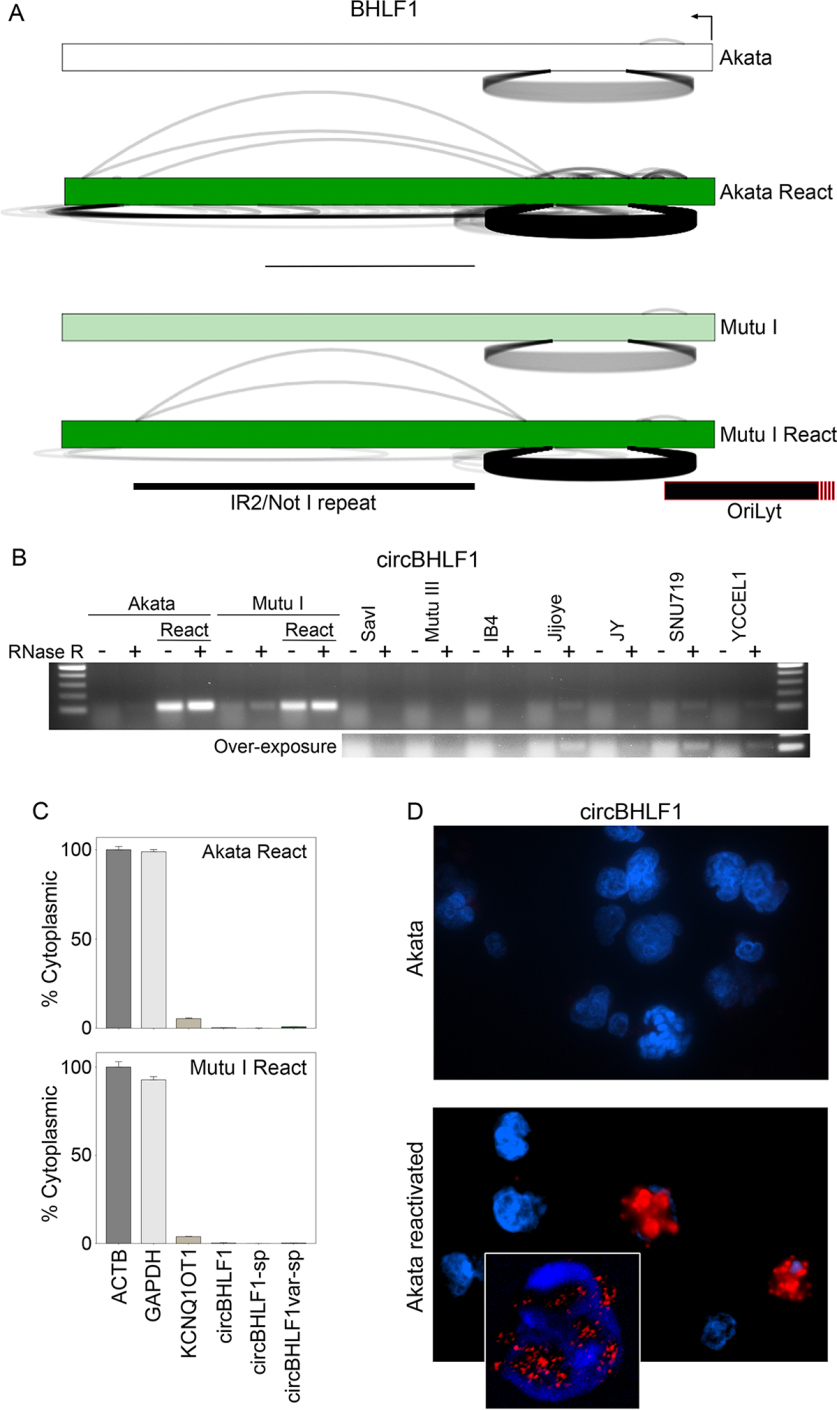
circBHLF1 detection in reactivated Akata and Mutu I cells. A) Backsplicing read counts (under arches) are derived from RNase R-seq datasets and forward splicing (over arches) and coverage data (exon color intensity) are derived from polyA-seq datasets. The number of arches (forward- and back-splicing) correspond to the number of junction spanning reads. Exon shading intensity reflects relative coverage levels across samples. B) RNase R-resistance of circBHLF1. All PCR reactions were repeated with similar results. C) Nuclear/cytoplasmic distribution of circBHLF1 using RT-qPCR. Cytoplasmically localized ACTB and nuclear localized KCNQ1OT1 are shown for comparison. Fraction cytoplasmic is relative to ACTB (cytoplasmic) and the most nuclear localized junction in the respective cell lines. Error bars represent standard deviations and were derived from triplicate qPCR reactions. These experiments were repeated once with similar results. Method for calculating fraction cytoplasmic is outlined in the methods section. D) BaseScope analysis of circBHLF1 cellular distribution. Inset shows confocal image of nuclear localization of circBHLF1.

BHLF1 is a single exon lytic gene that is expressed at low levels during latency but is induced to high levels during reactivation. In rough accordance with the expression profile of the parental BHLF1 gene, BHLF1 backsplicing was detected in most latency conditions and was found to be one of the most highly detected backsplices in the cell during reactivation (Figs 2 and 9 and S3 Fig). Using divergent primers to amplify across the backsplice junction, RNase R resistant backsplices were detected at high levels in reactivated Akata and Mutu cells and at lower levels in Jijoye, JY, SNU719 and YCCEL1 (Fig 9B). Sequencing of multiple PCR fragment clones from these amplified bands revealed not only the backsplice junction identified by find_circ but also a second, more frequently detected BHLF1 backsplice isoform derived from a non-canonical splice donor located 9 base pairs upstream from the circBHLF1 splice donor. During *in silico* validation of backsplicing (i.e. aligning sequence reads to the conjoined BHLF1 backsplices (mentioned above)), we also noted a high number of reads that similarly contained this 9 base splice donor shift (read counts not included in analysis of circBHLF1 in Fig 2 or S3 Fig). Realignment to conjoined backsplice junction sequences for this “alternate” backslice junction yielded 7310 junction spanning reads in reactived Akata cells, 3-fold more than the circBHLF1 backsplice reads in these conditions. To uniquely assess subcellular localization of each of these isoforms, we designed additional divergent primers with forward primers spanning either of the circBHLF1 and “circBHLF1-alt” backsplice junctions and a common reverse primer. Using these primers, the appropriate PCR fragments were amplified and circBHLF1 and circBHLF1alt were both found to display RNase R resistance (S4 Fig). Using these primers for RT-qPCR, both circular BHLF1 RNAs display predominantly nuclear localization (Fig 9C). Further, using BaseScope technology to specifically visualize localization of juxtaposed circBHLF1 backsplice junctions, predominantly nuclear localization was observed (Fig 9D). Together, this data indicates that these BHLF1 derived circular RNAs are abundant during reactivation, resistant to RNase R, and localize to the nucleus, suggesting possible nuclear functions. It is notable that circBHLF1 is located proximal to the lytic origin of replication (Fig 9A). Rennekamp and Lieberman [41] showed that a BHLF1 RNA associates with oriLyt DNA sequences through an R-loop configuration to facilitate lytic viral DNA replication. With the nuclear localization of circBHLF1 and circBHLF1alt and the cis proximity of their originating locus to oriLyt (Fig 9A), worth considering is the possibility that their closed circular structure could provide unique molecular features that engage with the replication complex and regulate lytic viral DNA replication.

## Discussion

Despite more than 50 years of study since the discovery of EBV, our appreciation for the extent and diversity of the EBV transcriptome has grown substantially over the past 15 years with findings of hundreds of new viral RNAs including EBV encoded microRNAs [42, 43], a viral (v)-snoRNA [4], stable intronic sequence (sis)RNAs [3] and scores of previously unknown polyadenylated and non-polyadenylated lytic transcripts [25, 27, 28, 34, 44]. Here we report that EBV expresses a repertoire of yet another class of RNAs, circRNAs, in both latency and reactivation. Many of the EBV encoded circRNAs are expressed at levels that are comparable to or higher than the majority of cellular encoded circRNAs (Fig 2B), supporting the contention of potential functional relevance. Some EBV circRNAs are expressed broadly across latency types (e.g. circRPMS1_E4_E3a, circRPMS1_E4_E2, and circEBNA_U) suggesting roles in fundamental processes during latent infection. Further, circRPMS1_E4_E3a and circRPMS1_E4_E2 were found to be expressed in two of two EBV positive stomach cancer biopsies tested, supporting *in vivo* relevance and possible roles in supporting the tumor phenotype. Though expressed during latency, the expression of circEBNA_W1_C1 and circEBNA_W2_C1 is restricted to type III latency and could be involved in type III latency specific processes such as facilitating Cp initiated transcript diversity, Cp promoter regulation, or other as yet unappreciated type III latency functions. circBHLF1 is detected in most latency cell models but displays extraordinarily high expression under reactivation conditions (S3 Fig). The linear form of BHLF1 is expressed at low levels during latency through the activity of an alternative latency promoter [45, 46]. Whether the observed expression of circBHLF1 in latency cell lines is due to transcription from the latency promoter and/or whether its expression derives from a small percentage of spontaneously reactivating cells is unclear at this time. Nevertheless, its high expression during reactivation, its proximity to OriLyt, and the known association of BHLF1 RNAs with OriLyt supports speculation that it could play a role in reactivation such as facilitating lytic DNA replication. Expression of circLMP2_E8_E2 displayed tissue specificity, being detected in both reactivation models tested but not in the type III latency cell lines, Jijoye and JY (with low levels detected in IB4). This data supports the involvement of *cis* and *trans* mechanisms that facilitate the regulation of LMP2 back-splicing and they support a potential unique role for circLMP2_E8_E2 in reactivation. Together, the findings reported here reveal a spectrum of EBV circRNAs with diverse expression profiles and likely unique roles in latent and lytic infection.

While most of the backspice junctions that we detected are located in latency gene loci and while several candidate viral circular RNAs were found to be expressed in the latency setting, it is notable that there are substantially more backsplice junctions detected during reactivation (Fig 2). During reactivation, there is substantial subversion of the cell transcription machinery by viral transcription leading to a predominance of viral transcripts in the cell [47]. Liang et al [48] showed that cell stress and resulting limiting of the splicing machinery can increase circular RNA formation relative to linear canonical splicing. This raises the possibility that reactivation might similarly stress the splicing machinery and induce circular RNA formation in this setting. An analysis of backsplicing to canonical splicing ratios of cellular genes, however, did not show a substantial induction of circular RNA formation during reactivation (S5 Fig). This suggests that the increase in viral backsplicing observed during reactivation may be due to virus related mechanisms rather than generalized stresses on the cell itself. This doesn’t preclude the possible involvement of local intra-cellular stresses on the splicing machinery during reactivation, however; for example in viral replication factories where there are high local concentrations of transcribing viral genomes. Even in this scenario, though, there must be *cis* specificity since not all spliced viral transcripts show backsplicing during reactivation (e.g. BZLF1, see S6 Fig). This supports the contention that backsplicing of viral genes during reactivation is regulated and that the induced expression of viral circular RNAs may have functional relevance. Notably, the lytic EBV BMLF1 protein has been shown to interact with splicing factors and to be involved in RNA transport, polyadenylation, splicing, and translation [49–54]. It will be important to determine whether BMLF1 plays a role in enhancing and/or regulating lytic backsplicing and viral circRNA formation. Further, with its known function in enhancing translation of some mRNAs, it will be interesting to assess whether BMLF1 potentially facilitates translation of cytoplasmically localized circRNAs such as circLMP2_E8_E2s.

One of the most notable functions of cellular circRNAs is the regulation of microRNA activity through sponging/sequestration mechanisms. Nevertheless, mining previously published PAR-CLIP data from seven EBV positive cell line models [37–40], we were unable to identify interactions between any viral or cellular microRNAs and EBV sequences spanning circular RNA exons. Since all of these studies were performed in latent cell systems, this does not preclude possible interactions between microRNAs and lytic EBV circRNAs, but does support the contention that the EBV circRNAs identified here serve other non-microRNA sponge functions.

While circRNAs are thought to mostly function through non-coding mechanisms, some have been shown to localize to ribosomes and to be translated [22–24]. Bencun et al [55] have previously performed ribosomal profiling of B-lymphocytes infected with the B95-8 and M81 strains of EBV to assess the translation of latent (B95-8 infection) and lytic (M81 infection) viral transcripts. Although circEBNA_U contains only a single 6-amino acid open reading frame (ORF), cells treated with harringtonine to map translation initiation sites showed a peak covering this short ORF [55]. Further, as noted in [55], the EBNA U exon contains a previously identified IRES that overlays this short ORF that is thought to regulate translation of downstream spliced EBNA reading frames [56]. In the circEBNA_U context, the IRES could conceivably self-regulate translation initiation of this ORF to generate a short functional peptide or to mediate some other regulatory function through an association with ribosomes.

Notably, circBHLF1 has the potential to code for a 200 amino acid ORF through two consecutive frame shifts at the backsplice junction (S7 Fig). Alignment of harringtonine/ribosomal RNA-seq data from B95-8 or M81 infected cells showed weak evidence of translation initiation at this site [55]. Although our RT-qPCR analysis showed nearly exclusive nuclear localization of circBHLF1, given the abundance of circBHLF1, it is possible that low levels of circBHLF1 could potentially associated with ribosomes and generate a translated product from this ORF.

While we observed minor harringtonine/ribosomal RNA-seq peaks at the initiation codon located at the beginning of exon 2 of LMP2, in the context of these infections, circLMP2 may not be expressed and/or any initiation at this site could represent initiation from linear LMP2B transcripts derived from the LMP2B promoter. Assessing whether circLMP2_E8_E2 is a novel isoform of LMP2B will require more detailed experiments to specifically assess circLMP2_E8_E2 association with ribosomes and perhaps epitope tagging of circLMP2_E8_E2.

Given the diverse spectrum of latency and lytic genes from which EBV circRNAs originate, their diverse patterns of expression and their different sub-cellular distributions, EBV circRNAs may play roles in a wide array of latent, lytic, nuclear and cytoplasmic functions in latency, reactivation, B-cells and epithelial cells in natural EBV infection and potentially in EBV-associated malignancies. Together, this study uncovers a new spectrum of virus encoded RNAs and should set the stage for new lines of research into virus biology and EBV-associated lymphoma and epithelial cancers. In particular, the finding of latency associated circRNAs adds to the repertoire of potential future therapeutic targets. Further, with their increased stability compared to linear RNAs, circRNAs are receiving attention as potential liquid biopsy markers [20, 57]. With antibodies to EBV antigens previously identified as predictors of nasopharyngeal carcinoma (e.g. [58–60]), serum viral circRNAs could someday show predictive and/or diagnostic potential in EBV associated disorders.

## Materials and methods

### Cell culture

Akata (obtained from Kenzo Takada), Mutu I, Sav I, Mutu III, IB4, Jijoye, JY (Mutu I, Sav I, Mutu III, IB4, Jijoye, and JY cell lines obtained from the laboratory of Samuel H Speck) and SNU719 (Korean Cell Line Bank) cells were cultured in RPMI 1640 media (Fisher Scientific, catalog no. SH30027) plus 10% fetal bovine serum (FBS) (Thermo Fisher, catalog no. 10437). YCCEL-1 (Korean Cell Line Bank) cells were grown in Eagle's minimum essential medium (EMEM) (ATCC, catalog no. 30–2003) supplemented with 10% FBS. All cells were cultured at 37°C in a 5% CO_2_ incubator.

### Induction of EBV reactivation

Akata and Mutu I cells were spun down and resuspended at a concentration of 10^6^ cells/ml in fresh RPMI 1640 medium (10%FBS). Anti-human IgG (Sigma-Aldrich, catalog no. I5260) or anti-human IgM (Sigma-Aldrich, catalog no. I0759) was added to Akata and Mutu I cell suspensions, respectively, to a final concentration of 10 ug/ml. Treated and untreated cells were harvested 24 h and 48 h later for Akata and Mutu I cells, respectively, and subjected to RNA isolation.

### RNA preparations

Whole cell RNA preparations were carried out using TRIzol reagent (Thermo Fisher, catalog no. 15596) according to the vendor’s recommended protocol. For tumor and normal tissue, pieces were first ground finely using a mortar and pestle in liquid nitrogen prior to disruption with TRIzol reagent. Nuclear and cytoplasmic RNAs were isolated using the Cytoplasmic & Nuclear RNA Purification Kit from Norgen Biotek Corp. (catalog no. 21000) according to the vendor’s protocol. All RNA preparations were subjected to DNase treatment twice using the DNA-free kit (Thermo Fisher, catalog no. AM1906).

### RNA-sequencing

RNA-sequencing was performed at the Beijing Genomics Institute (BGI). For polyA-seq, RNAs were selected using a poly dT column, and for ribodepletion-seq ribosomal RNAs were excluded using hybrid capture for 28S, 18S and 5S ribosomal RNAs. For RNase R-seq, RNAs were subjected to DNA and rRNA depletion, followed by linear RNA depletion using RNase R. For all sequencing, Truseq stranded libraries were generated and sequenced using 2x100 base sequencing on a HiSeq 4000 system.

### Backsplice junction analysis

Back-splicing was analyzed using find_circ [26] with default parameters. For *in silico* validations, a STAR [61] genome index was generated which contains the human hg38 genome build plus conjoined backsplice junctions for each candidate EBV backsplice identified by find_circ. Raw fastq files were aligned to this combined genome index using STAR (–outFilterMultimapNmax 20 –outSAMtype BAM SortedByCoordinate–outWigType wiggle–outWigNorm None) and reads spanning EBV backsplice junctions with a minimum of 12 base overlap (minimum of 90% homology) on each side of the junction were pulled out for visualization on the Integrative Genomics Viewer (IGV) and number of reads mapping to each junction in each cell line were quantified for reporting.

Notably, while intron lariats are similarly covalently closed RNAs and can be enriched by RNase R treatment, lariat RNAs are typically unstable due to the action of debranching endonucleases that target the 5’ to 2’ junction. In addition, reverse transcriptase displays resistance crossing the 5’ to 2’ junction, thereby further under-representing lariat junction reads in RNA-seq data. Further, all EBV backsplice junctions occurred at canonical GU AG splice donor-acceptor motifs and showed no evidence of micro–insertions/-deletions or single nucleotide substitutions that are typically observed following reverse transcription across lariat junctions. We therefore conclude that all 33 EBV backsplice junctions reported here likely represent true circRNAs.

### Splice junction and expression exon structure plots

For canonical splicing and coverage display, RNA-seq data from polyA libraries were analyzed by STAR alignment against the human hg38 plus chrEBV_Akata_inverted genome using STAR (–outFilterMultimapNmax 20 –outSAMtype BAM SortedByCoordinate–outWigType wiggle–outWigNorm None). Splice junction data from .SJ files and wiggle output files were used to generate junction read numbers and coverage information. Backsplice read counts were extracted from .bed junction count files derived from find_circ output. For visualization of forward splicing, coverage and backsplicing, in house software (circleVis) was developed. Exon level coverage was represented by color intensity with canonical and backsplice junction curves plotted above and below the exon diagram, respectively. Each junction count was individually plotted.

For visualization of splicing and coverage across the EBNA locus, a custom analysis pipeline was developed to accommodate unique problems associated with aligning to repetitive elements. Specifically, all canonical forward splice junctions were quantified from STAR alignments to an artificial mini EBV genome containing upstream unique sequences, two copies of BamHI W repeats, and downstream unique regions extending past the EBNA1 gene. Coverage and backsplice information was generated using STAR (coverage) and find_circ (backsplice counts) alignments to an artificial mini EBV genome containing one copy of the BamHI W repeats inserted between unique upstream and downstream EBNA locus sequences. For display, forward spliced read counts, coverage and backsplice read counts associated with the W1 and W2 exons were divided by the appropriate number of exons/junctions to distribute these counts equivalently across 7 W repeats.

### RNase R resistance analysis

5 ug of total RNA was incubated with or without 20 units of RNase R (Applied Biological Materials, Inc., catalog no. E049). Briefly, no (control) or 1.5 ul RNase R (30u) (test) and 3 ul of 10X RNase R buffer were added to 5 ug of RNA in a total volume of 30 ul and incubated in a 37C water bath for 30 minutes. Either no (control) or 1.5 ul (30 units) (test) more RNase R was added to reactions and incubated for an additional 1.5 hours in a 37C water bath. RNAs were cleaned and concentrated using the RNA Clean & Concentrator-5 kit (Zymo Research, catalog no. R1015) and eluted in 10ul H_2_O for use in PCR reactions.

### RT-PCR

cDNA was synthesized from total RNA (control or RNase R treated where indicated) using SuperScript IV First-strand Synthesis System (Thermo Fisher, catalog no. 18091) and the cDNAs were amplified by taq-PCR (Thermo Fisher, catalog no. 11304) following the vendor’s protocol. PCR products were run on a 1.5% agarose gel at 4°C. PCR products were cut out and purified using the NucleoSpin Gel & PCR Clean-up Kit (Clontech, catalog no. 740609). The resulting PCR fragments were cloned into the pCR4-TOPO vector (Thermo Fisher, catalog no. 450030) and the inserts were Sanger sequenced.

### RT-qPCR

For assessing nuclear/cytoplasmic localization, cDNA was synthesized from total RNA using SuperScript IV First-strand Synthesis System (Thermo Fisher, catalog no. 18091) according to the manufacturer’s protocol. qPCR analysis was performed using iQ SYBR Green Supermix (Bio-Rad, catalog no. 170–8882) on a Bio-Rad CFX96 instrument as follows: 1 μl of cDNA and 1 μl of 10 μM primers were mixed with 10 μl of SYBR green supermix and 8 μl nuclease-free H2O to a 20 μl reaction volume. Polymerase was activated and cDNA was denatured at 95°C for 5 minutes. cDNA was then amplified for 40 cycles with 15 s denaturation at 95°C, 60s annealing/extension and plate reading at 60°C. Melting curve analysis was performed at temperatures from 60°C to 90°C with 0.5°C increment per 5 s. Expression fold changes were calculated using the comparative CT method (2-ΔΔCT). Cytoplasmic enrichment was calculated as 2^(((test gene (Ctnuclear—Ct_cytoplasmic_))–(nuclear reference gene (Ctnuclear—Ct_cytoplasmic_)))/((cytoplasmic reference gene (Ctnuclear—Ct_cytoplasmic_))–(nuclear reference gene (Ctnuclear—Ct_cytoplasmic_))).

### PCR primers

ACTB forward:                                        5’- CGTCATACTCCTGCTTGCTG

ACTB reverse:                                        5’- GGCATCCTCACCCTGAAGTA

ACTB (qPCR) forward:                          5’- CACTCTTCCAGCCTTCCTTC

ACTB (qPCR) reverse:                           5’- GTACAGGTCTTTGCGGATGT

GAPDH exon 1 forward:                        5’- AAGGTGAAGGTCGGAGTCAAC

GAPDH exon 2 reverse:                        5’- GGGTCATTGATGGCAAC

KCNQ1OT1 forward:                           5’- TACCGGATCCAGGTTTGCAGTACA

KCNQ1OT1 reverse:                            5’- GCTGATAAAGGCACCGGAAGGAAA

circEBNA exon W1 (Akata) forward:    5’- GGGAGACCGAAGTGAAGTCC

circEBNA exon W1 (B95-8) forward:    5’- GGGAGACCGAAGTGAAGGCC

circEBNA exon C1 (W1) reverse:          5’- ATGGTAAGAACCCTGCGATG

circEBNA exon W1-C1-sp forward       5’- TATCGGGCCAGAGATGGCAT

circEBNA exon C1 reverse (W1-C1-sp) 5’- TAGATGATTTGCGGGTTACATGA

circEBNA exon W2 forward:                 5’- AGAACCCAGACGAGTCCGTA

circEBNA exon C1 (W2) reverse:         5’- CATGGTAAGAACCCTGCGAT

circRPMS1 exon 4 forward:                  5’- CTAGTGCTGCATGGGCTCCT

circRPMS1 exon 3a reverse:                5’- GTCATACGCCCGTATTCACA

circRPMS1 exon 4-3a-sp forward:        5’-GCTGTTCCTGAACGACGAG

circRPMS1 exon 4-3a-sp reverse:        5’- ACACGCCGGACCTTGCC

circBHLF1 forward:                            5’- CCAGAGGAGCCCCAGAAC

circBHLF1var-sp forward:                  5’- CCCAGAACCAGGTGCACC

circBHLF1-sp forward:                      5’- AGGCAAGCCGGGTGCAC

circBHLF1 reverse:                            5’- ATGCTGCATCCGCTAGTCC

LMP2A exon 1 forward:                      5’- CTACTCTCCACGGGATGACTC

LMP2A exon 2 reverse:                      5’- AGGTAGGGCGCAACAATTAC

circLMP2 exon 4 forward:                 5’- TTCTGGTGATGCTTGTGCTC

circLMP2 exon 5 forward:                 5’- TCACTGATTTTGGGCACACTT

circLMP2 exon 6 forward:                 5’- ATCGCTGGTGGCAGTATTTT

circLMP2 exon 7 forward:                 5’- GCTCTCGCACTCTTGTTGCT

circLMP2 exon 8 forward:                 5’- TCATTAGATGCTGCCGCTAC

circLMP2 exon 2 reverse:                 5’- AGGTAGGGCGCAACAATTAC

To assess expression across cell lines, cDNA was synthesized using SuperScript IV First-strand Synthesis System (Thermo Fisher, catalog no. 18091) according to the manufacturer’s protocol. qPCR analysis was performed using TaqMan Fast Advanced Master Mix (Thermo Fisher, catalog no. 4444557) on a Bio-Rad CFX96 instrument as follows: 1 μl cDNA and 1 μl 20X TaqMan assay were mixed with 10 μl of 2X TaqMan Fast Advanced Master Mix and 8 μl nuclease-free H2O to a 20 μl reaction volume. After a 2 minute UNG incubation at 50°C, polymerase was activated at 95°C for 2 minutes. cDNA was then denatured and amplified/extended for 40 cycles with a 3 s denaturation at 95°C and a 30 s annealing/extension and plate reading at 60°C or 66°C. The TaqMan assays were designed using the following primers, with the probe primers designed to hybridize to 13 bases on each side of the backsplice junction:

circRPMS1_E4_E3a:

circRPMS1 exon 4 forward:                                   5’-CTAGTGCTGCATGGGCTCCT

circRPMS1 exon 3a reverse:                                  5’-GTCATACGCCCGTATTCACA

circRPMS1_E4_E3a (Probe (fluorescein)):            5’-gtcgacgggcaaGGtccggcgtgtcc

BHLF1:

circBHLF1 forward:                                             5’- CCAGAGGAGCCCCAGAAC

circBHLF1 reverse:                                              5’- ATGCTGCATCCGCTAGTCC

circBHLF1 (Probe (fluorescein)):                          5’-ccaggcaagccgGGtgcaccggacca

EBNA-U:

circEBNA_U forward:                                         5’-TCTGGAGCCTGACCTGTGA

circEBNA_U reverse:                                          5’-TCTCTGGGCTGCAGAATCA

circEBNA_U Probe (fluorescein):                         5’-cgtctcctttaaGGtaacttaggaag

RPL30 (reference gene):

RPL30 forward:                                                   5’-CACCAGTTTTAGCCAACATAGC

RPL30 reverse:                                                    5’-TGAAGATGATCAGACAAGGCAA

RPL30 (Probe (fluorescein)):                                5’-TTCTCGCTAACAACTGCCCAGCT

Relative expression was calculated using the delta delta Ct method using RPL30 as the reference gene and uninduced Akata cells as the reference sample.

### BaseScope assay

Cell pellet preparation was carried out in accordance with the vendor’s protocol (Advanced Cell Diagnostics). Specifically, Akata cells were cultured with or without 10 μg/ml of anti-human IgG (Sigma-Aldrich, cat # I5260). Twenty-four hours post treatment, cells were harvested and washed once with 1X phosphate-buffered saline (PBS). Cell pellets were resuspended in 10ml of 10% neutral buffered formalin (Sigma-Aldrich, Cat # HT501128) with 40 ml 1X PBS and fixed at room temperature for 24 h. Cells were washed twice with 10ml of 1X PBS and the cell pellets were resuspended in liquified HistoGel (ThermoFisher, Cat # HG-4000-012) and solidified on ice. Cell blocks were then paraffin embedded and 5 μm sections were mounted onto Superfrost plus slides (Fisher, Cat # 4951PLUS).

BaseScope assays were performed in accordance with the BaseScope Reagent Kit-RED protocol (Advanced Cell Diagnostics, Cat # 322970) using a circBHLF1 backsplice specific probe (Advanced Cell Diagnostics, Cat # BA-V-EBV-BHLF1-circRNA). Assays were performed at room temperature unless otherwise indicated. Sections were baked at 60°C for 1 h, followed by deparaffinizing in xylene twice for 5 min, ethanol twice for 5 min, and baked at 60°C for 5 min. Slides were first treated with hydrogen peroxide for 10 min at RT, treated with target retrieval reagent for 15 min at 100°C, and treated with protease III for 15 min at 40°C, with two distilled water rinses between each treatment. BaseScope probes were then applied and incubated for 2 h at 40°C in a HybEZ oven followed by incubation with reagents AMP0 for 30 min at 40°C, AMP1 for 15 min at 40°C, AMP2 for 30 min at 40°C, AMP3 for 30 min at 40°C, AMP4 for 15 min at 40°C, AMP5 for 30 min at RT and AMP6 for 15 min at RT. Slides were rinsed twice with wash buffer for 2 min between each AMP incubation. Slides were incubated with Fast Red for 10 min at RT in the dark, then counterstained with Gill’s hematoxylin (Sigma-Aldrich, Cat # GHS132) for 15 min at 60°C and mounted in ProLong Gold Antifade Mountant with DAPI (ThermoFisher Scientific, Cat # P36941). The BaseScope Fast Red and DAPI signals were visualized on an Eclipse Ti2 inverted microscope system (Nikon).

## Supporting information

S1 Fig Integrative Genome Viewer (IGV) display of backsplice junctions identified in ribodepletion-seq and RNase R-seq analysis of reactivated Akata cells. (JPG)

S2 Fig Relative expression of circRPMS1_E4_E3a, circBHLF1, and circEBNA_U across cell lines. RT-qPCR was performed using a TaqMan assay with probes spanning the backsplice junctions. Presented data is representative experiment from two separate experiments and error bars represent standard deviation from triplicate qPCR reactions for each sample. Data is presented as delta delta Ct values with RPL30 as reference gene and uninduced Akata cells as reference condition.
(JPG)

S3 Fig Graphical presentation of splicing and exon specific coverage of the BHLF1 gene. Backsplicing read counts (under arches) are derived from RNase R-seq datasets and forward splicing (over arches) and coverage data (exon color intensity) are derived from polyA-seq datasets. The number of arches (forward- and back-splicing) correspond to the number of junction spanning reads. Exon shading intensity reflects relative coverage levels across samples. Proximal lytic origin of replication (OriLyt) shown in black.
(JPG)

S4 Fig RNase R-resistance of circBHLF1 and circBHLF1alt circular RNAs. Divergent primers with forward primers specific to the backsplice junction of circBHLF1 and circBHLF1alt and a common reverse primer, were used to detect the respective circular RNA species (results verified by sequencing). These experiments were repeated with similar results.
(JPG)

S5 Fig Percent of backsplice reads to total splice reads across cellular genes in ribodepleted RNA-seq data in Akata, reactivated Akata, Mutu I and reactivated Mutu I cells. (JPG)

S6 Fig Graphical presentation of splicing and exon specific coverage of the BZLF1 gene. Backsplicing read counts (under arches) are derived from RNase R-seq datasets and forward splicing (over arches) and coverage data (exon color intensity) are derived from polyA-seq datasets. The number of arches (forward- and back-splicing) correspond to the number of junction spanning reads. Exon shading intensity reflects relative coverage levels across samples.
(JPG)

S7 Fig Longest reading frame of circBHLF1. (JPG)

S1 Table Listing of all ribodepletion-seq junction read counts detected using find_circ and comparison of to the number of junction read counts derived from RNase R-seq for each respective junction. (XLSX)

S2 Table Junction spanning read counts (minimum 12 base overhang and 90% homology) derived from realignment to conjoined backsplice junctions. Note that some of the less abundant backsplices (e.g. several of the RPMS1 and the circBHRF1 backsplice junctions) met the 5 read minimum threshold in RNase R sequencing of nuclear and/or cytoplasmic fractions but show less than 5 reads in any whole cell sequencing data displayed here.
(XLSX)

S3 Table Counts and ranking across RNase R datasets. (XLSX)

S1 File *In silico* assessment/validation of backsplice junctions identified across datasets using find_circ. Reads from each RNase R-seq dataset were aligned to conjoined backsplice junctions using STAR and displayed (squished) on the Integrative Genome Viewer (IGV). Shown for each junction are the dataset with the maximal junction spanning read counts.
(PDF)
