# Supplementary figures and images for: The Epstein Barr virus circRNAome
Source: PLoS Pathog. 2018 Aug 6;14(8):e1007206. doi: 10.1371/journal.ppat.1007206 (PMC6095625; doi:10.1371/journal.ppat.1007206)

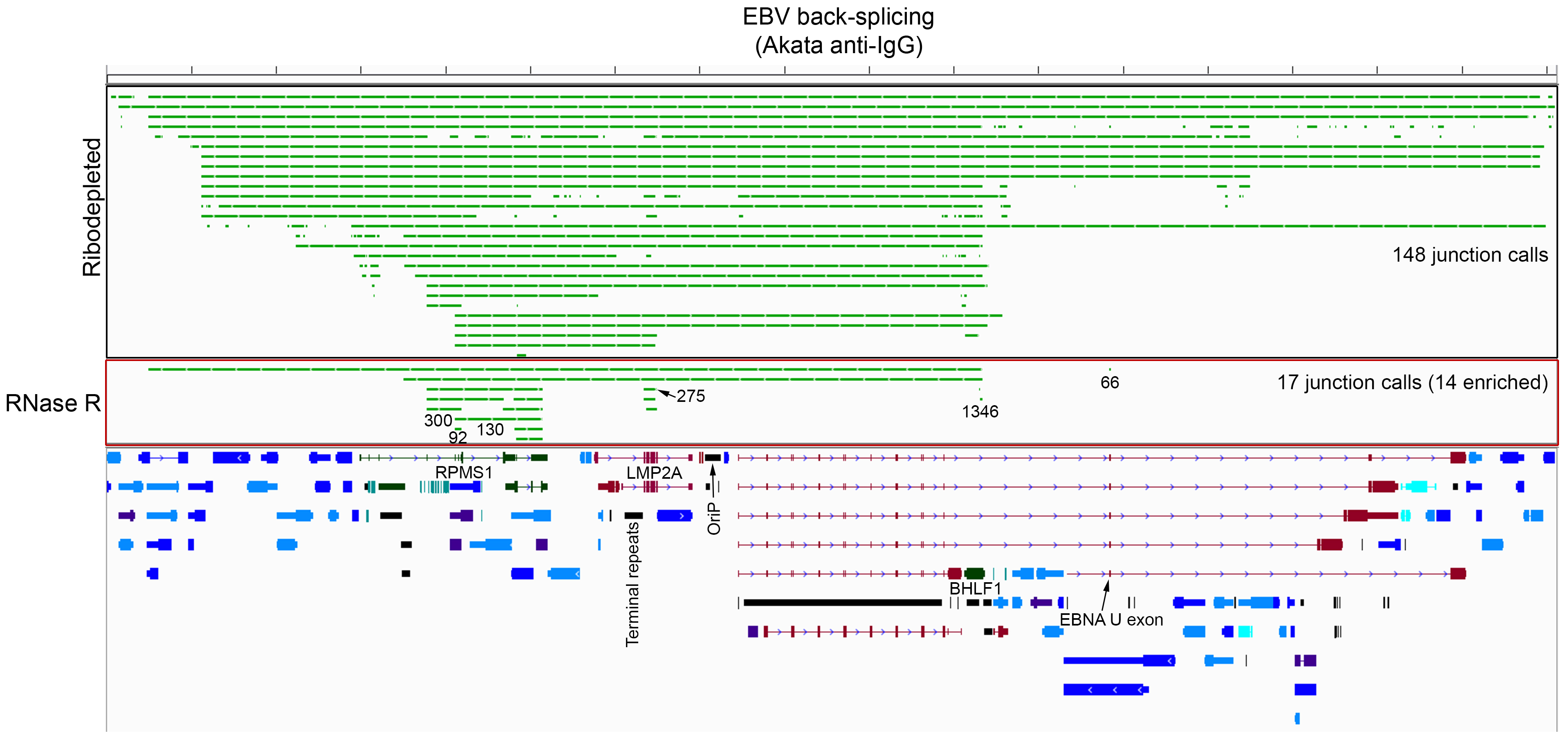

Supplement: S1 Fig — (JPG) [file ppat.1007206.s001.jpg]

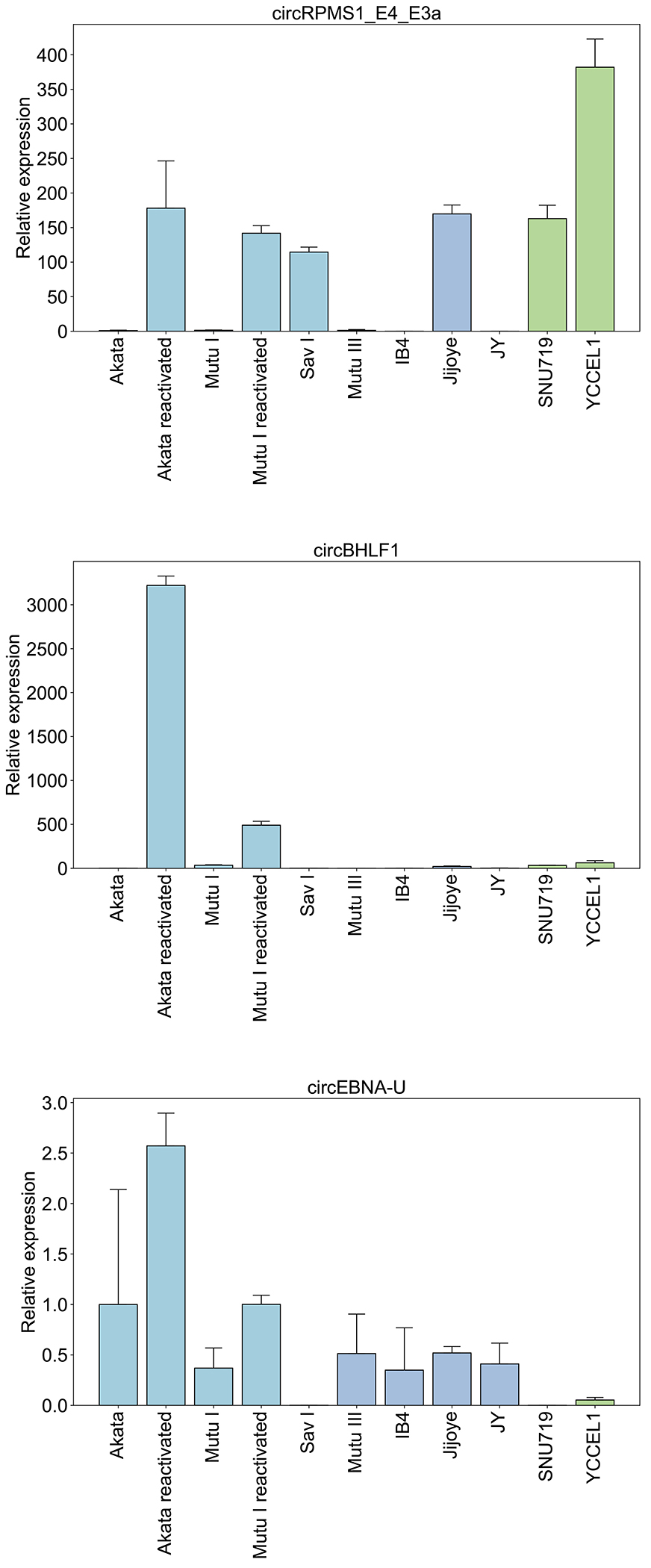

Supplement: S2 Fig — RT-qPCR was performed using a TaqMan assay with probes spanning the backsplice junctions. Presented data is representative experiment from two separate experiments and error bars represent standard deviation from triplicate qPCR reactions for each sample. Data is presented as delta delta Ct values with RPL30 as reference gene and uninduced Akata cells as reference condition. (JPG) [file ppat.1007206.s002.jpg]

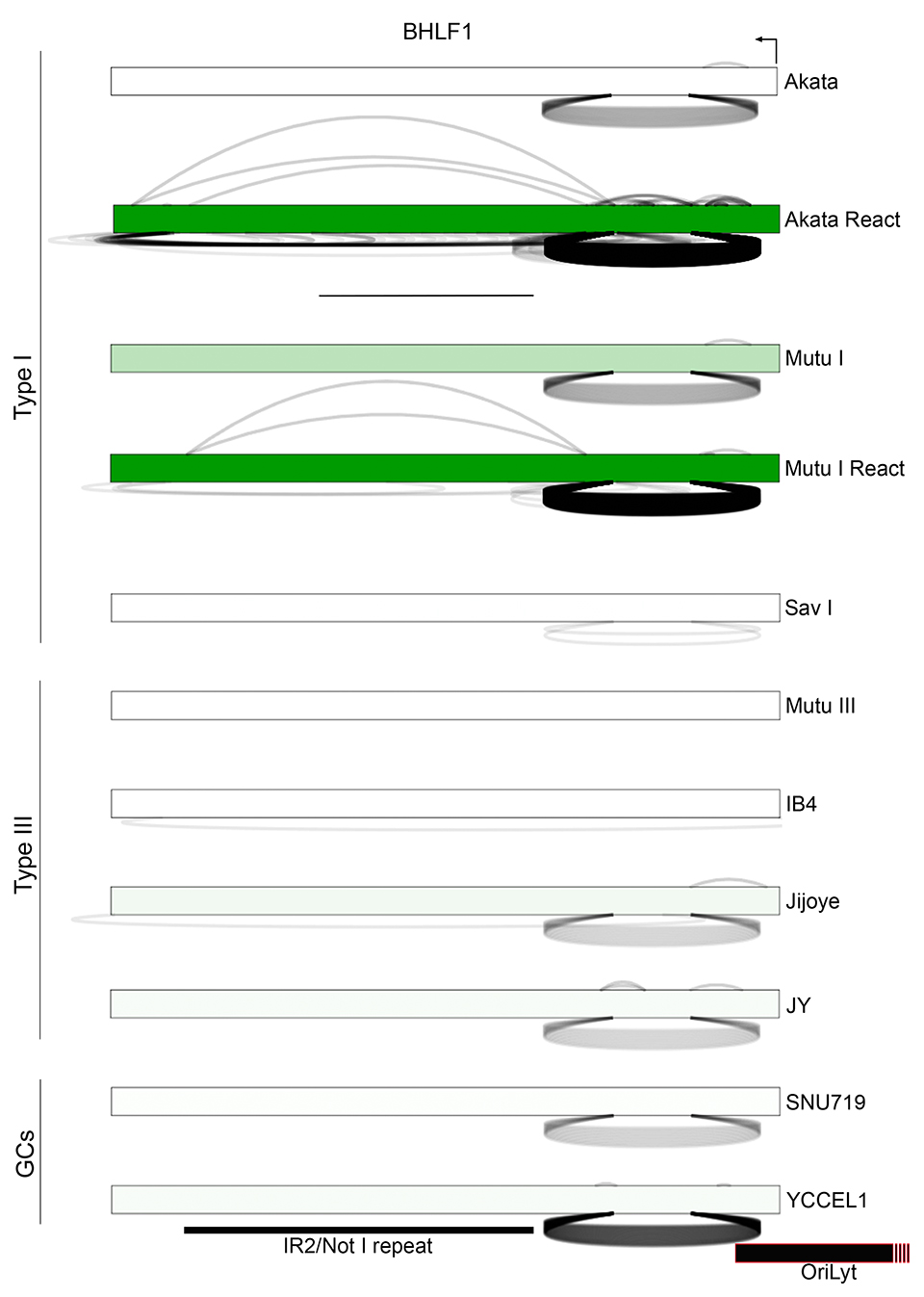

Supplement: S3 Fig — Backsplicing read counts (under arches) are derived from RNase R-seq datasets and forward splicing (over arches) and coverage data (exon color intensity) are derived from polyA-seq datasets. The number of arches (forward- and back-splicing) correspond to the number of junction spanning reads. Exon shading intensity reflects relative coverage levels across samples. Proximal lytic origin of replication (OriLyt) shown in black. (JPG) [file ppat.1007206.s003.jpg]

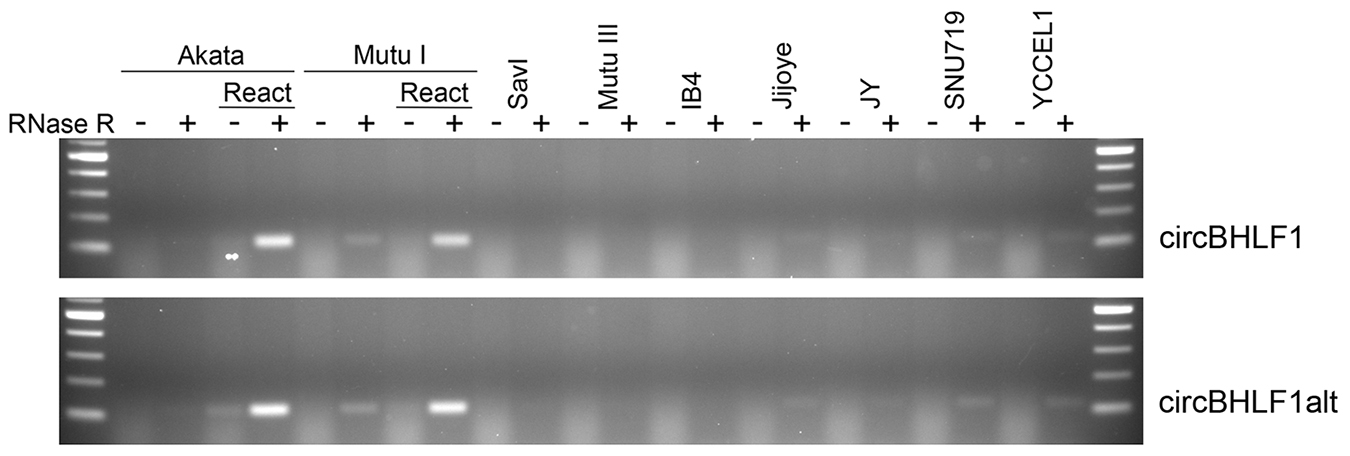

Supplement: S4 Fig — Divergent primers with forward primers specific to the backsplice junction of circBHLF1 and circBHLF1alt and a common reverse primer, were used to detect the respective circular RNA species (results verified by sequencing). These experiments were repeated with similar results. (JPG) [file ppat.1007206.s004.jpg]

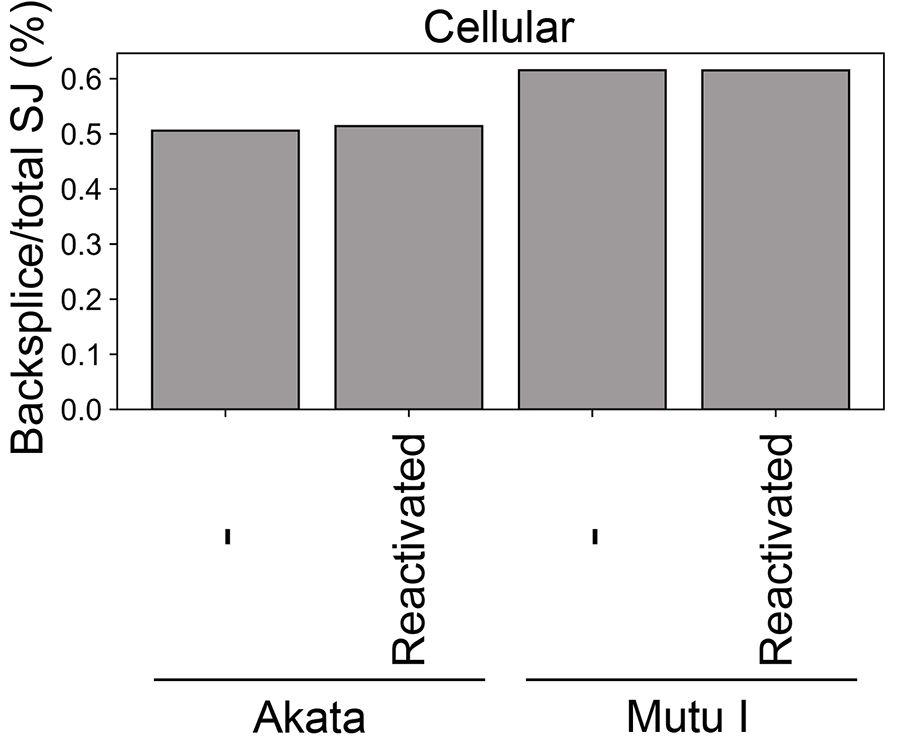

Supplement: S5 Fig — (JPG) [file ppat.1007206.s005.jpg]

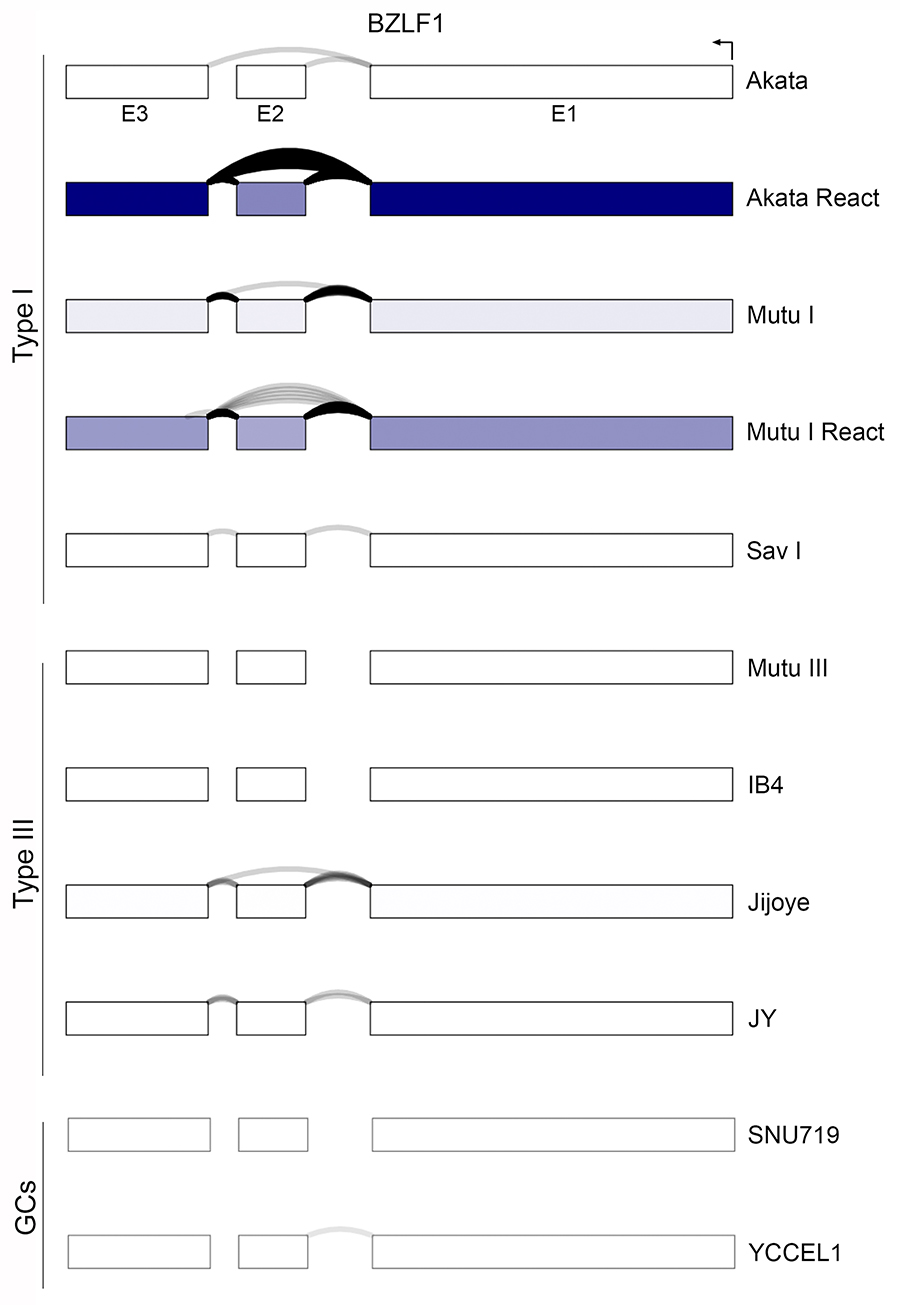

Supplement: S6 Fig — Backsplicing read counts (under arches) are derived from RNase R-seq datasets and forward splicing (over arches) and coverage data (exon color intensity) are derived from polyA-seq datasets. The number of arches (forward- and back-splicing) correspond to the number of junction spanning reads. Exon shading intensity reflects relative coverage levels across samples. (JPG) [file ppat.1007206.s006.jpg]

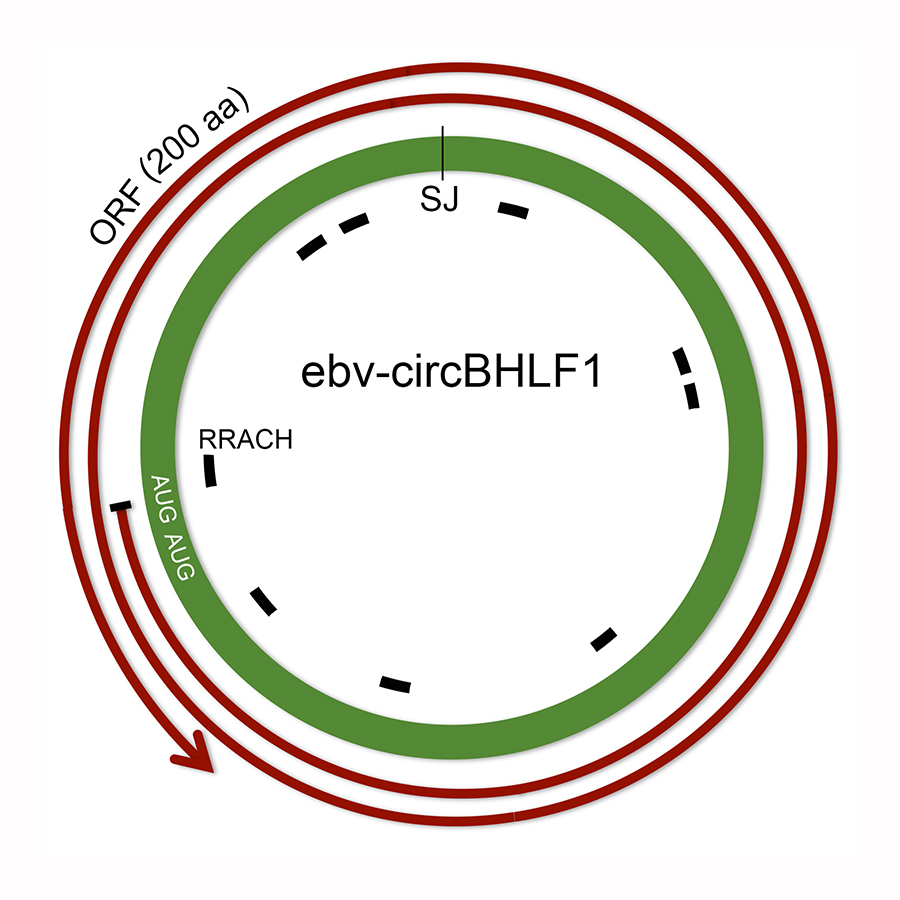

Supplement: S7 Fig — (JPG) [file ppat.1007206.s007.jpg]
