# Supplementary material for: The Epstein Barr virus circRNAome
Source: PLoS Pathog. 2018 Aug 6;14(8):e1007206. doi: 10.1371/journal.ppat.1007206 (PMC6095625; doi:10.1371/journal.ppat.1007206)

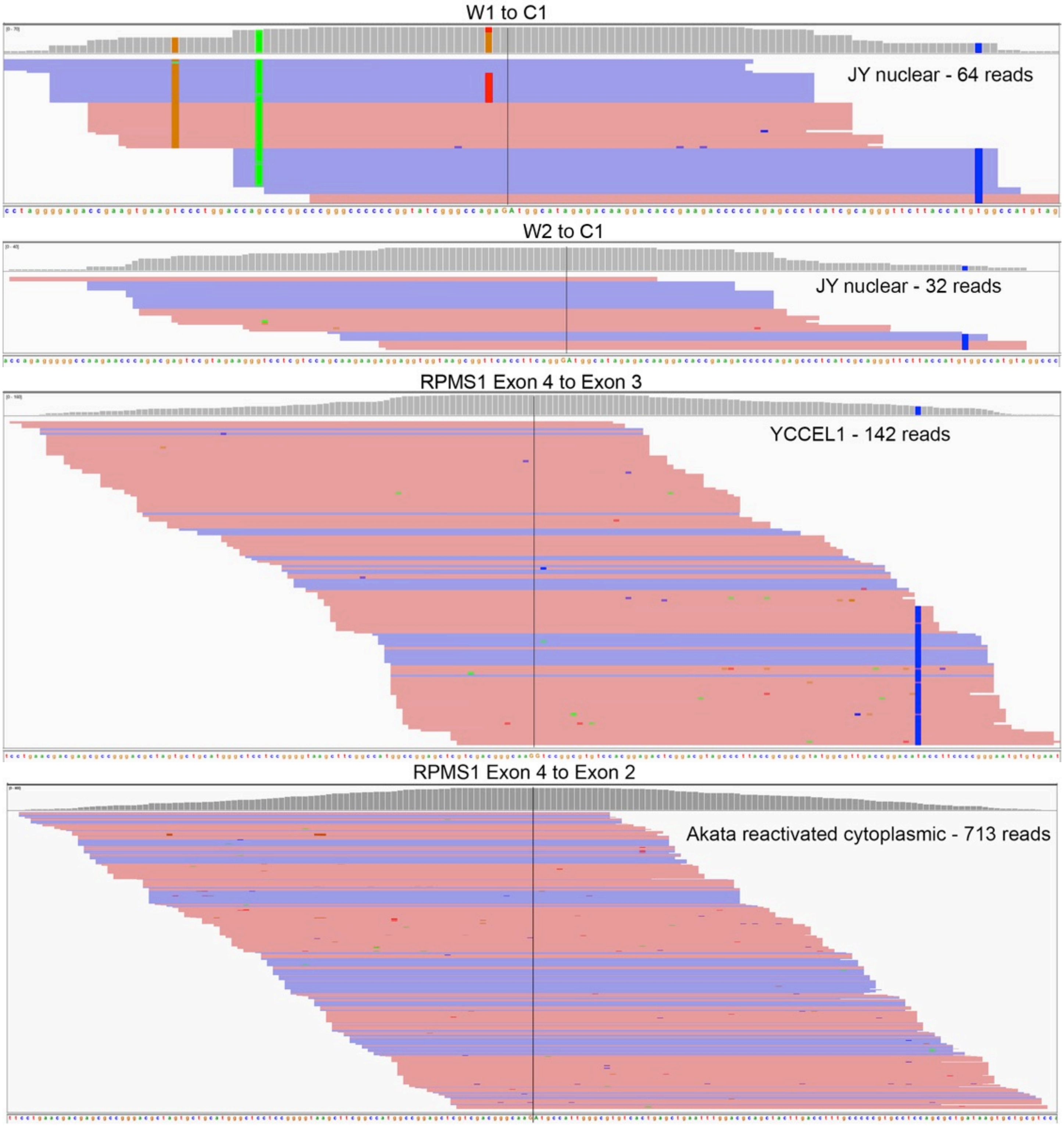
W1 to C1
JY nuclear - 64 reads
W2 to C1
JY nuclear - 32 reads
RPMS1 Exon 4 to Exon 3
YCCEL1 - 142 reads
RPMS1 Exon 4 to Exon 2
Akata reactivated cytoplasmic - 713 reads

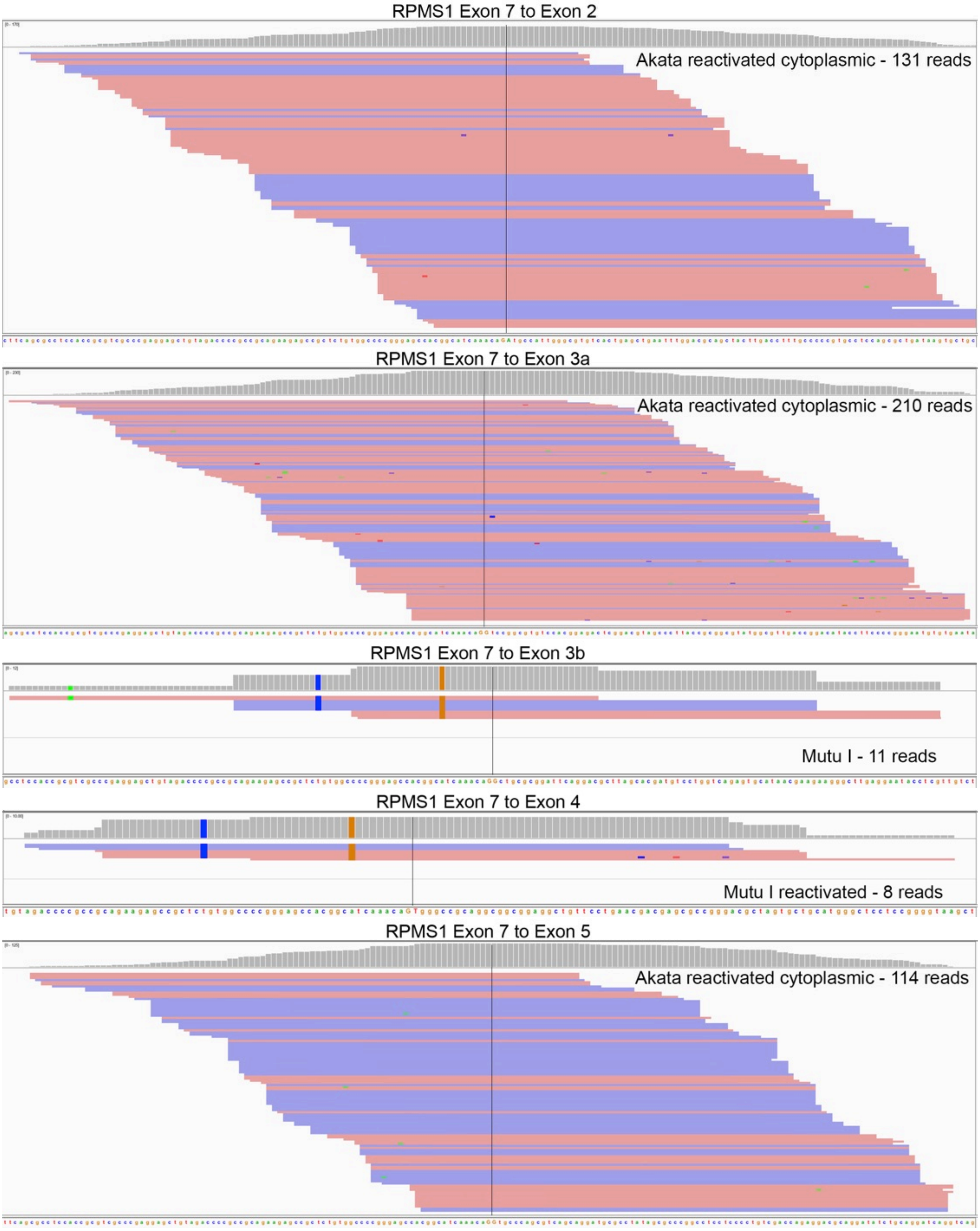
RPMS1 Exon 7 to Exon 2
Akata reactivated cytoplasmic - 131 reads
RPMS1 Exon 7 to Exon 3a
Akata reactivated cytoplasmic - 210 reads
RPMS1 Exon 7 to Exon 3b
Mutu I - 11 reads
RPMS1 Exon 7 to Exon 4
Mutu I reactivated - 8 reads
RPMS1 Exon 7 to Exon 5
Akata reactivated cytoplasmic - 114 reads

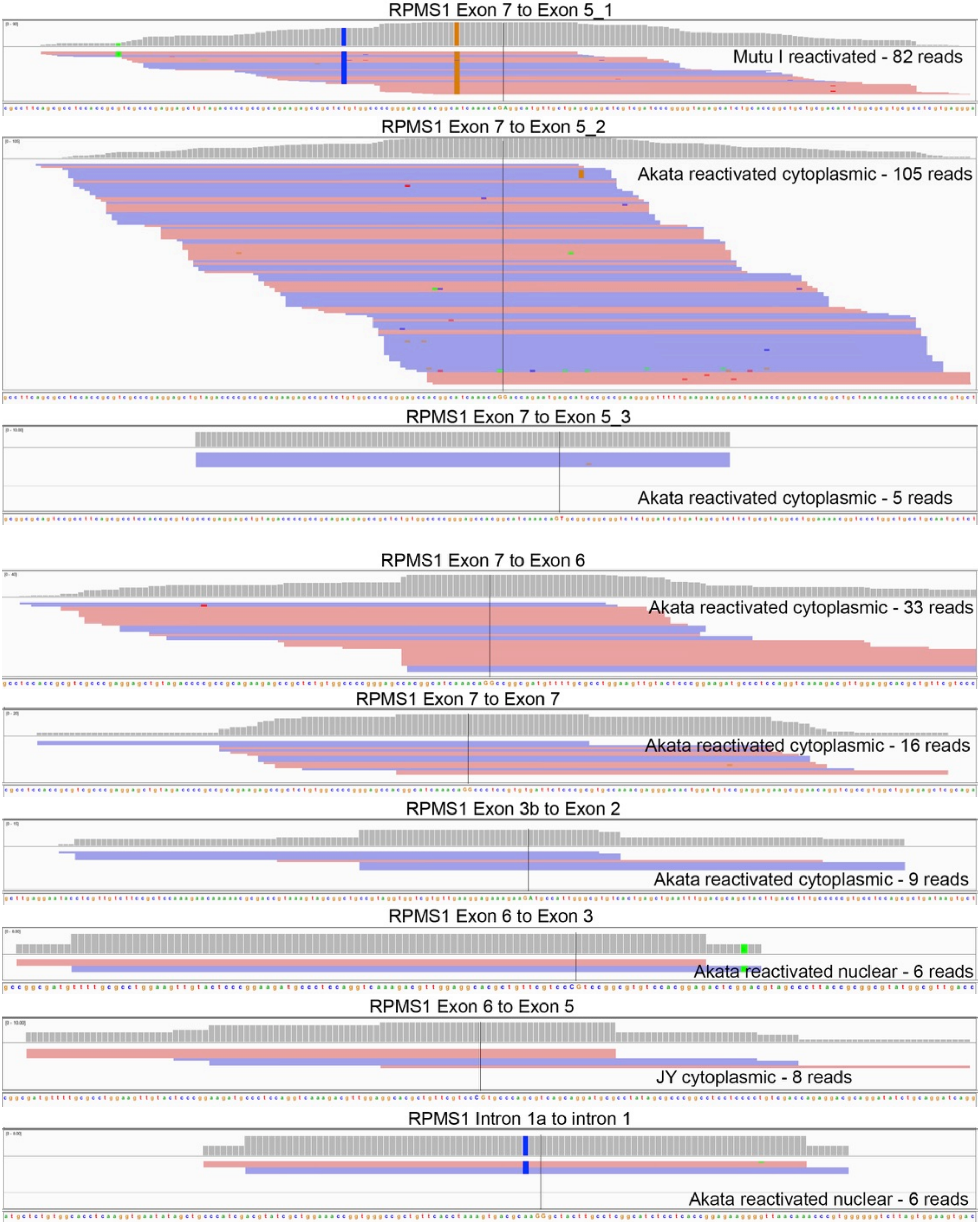
RPMS1 Exon 7 to Exon 5_1
Mutu I reactivated - 82 reads
RPMS1 Exon 7 to Exon 5_2
Akata reactivated cytoplasmic - 105 reads
RPMS1 Exon 7 to Exon 5_3
Akata reactivated cytoplasmic - 5 reads
RPMS1 Exon 7 to Exon 6
Akata reactivated cytoplasmic - 33 reads
RPMS1 Exon 7 to Exon 7
Akata reactivated cytoplasmic - 16 reads
RPMS1 Exon 3b to Exon 2
Akata reactivated cytoplasmic - 9 reads
RPMS1 Exon 6 to Exon 3
Akata reactivated nuclear - 6 reads
RPMS1 Exon 6 to Exon 5
JY cytoplasmic - 8 reads
RPMS1 Intron 1a to intron 1
Akata reactivated nuclear - 6 reads

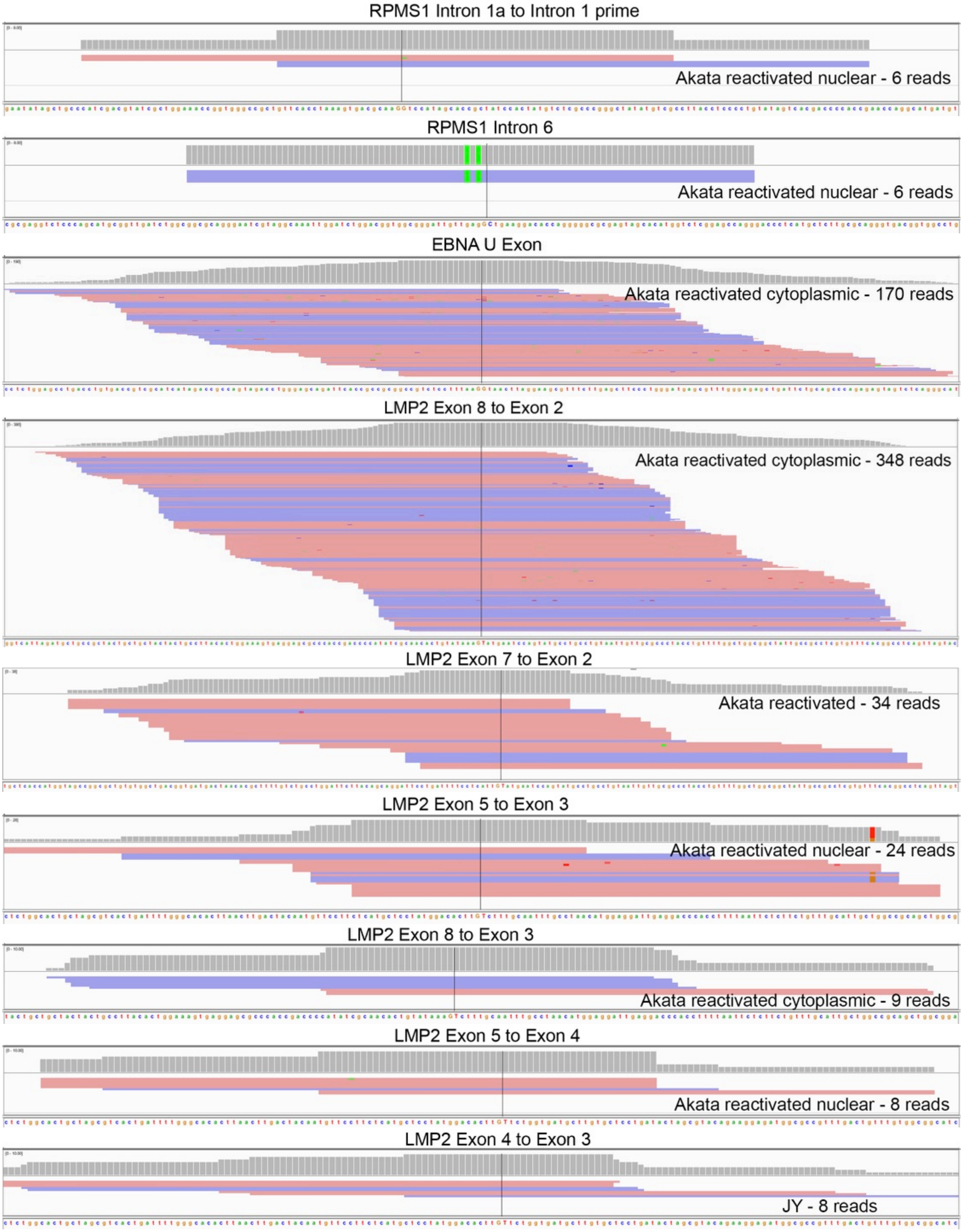
RPMS1 Intron 1a to Intron 1 prime
Akata reactivated nuclear - 6 reads
RPMS1 Intron 6
Akata reactivated nuclear - 6 reads
EBNA U Exon
Akata reactivated cytoplasmic - 170 reads
LMP2 Exon 8 to Exon 2
Akata reactivated cytoplasmic - 348 reads
LMP2 Exon 7 to Exon 2
Akata reactivated - 34 reads
LMP2 Exon 5 to Exon 3
Akata reactivated nuclear - 24 reads
LMP2 Exon 8 to Exon 3
Akata reactivated cytoplasmic - 9 reads
LMP2 Exon 5 to Exon 4
Akata reactivated nuclear - 8 reads
LMP2 Exon 4 to Exon 3
JY - 8 reads

LMP2 Exon 1 to RPMS1 Exon 3

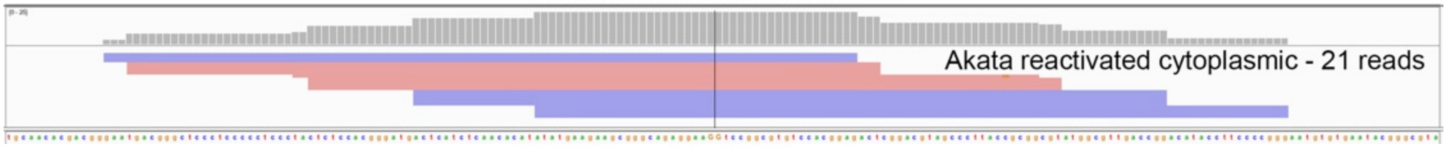

LMP2 Exon 1 to A73 Exon 3

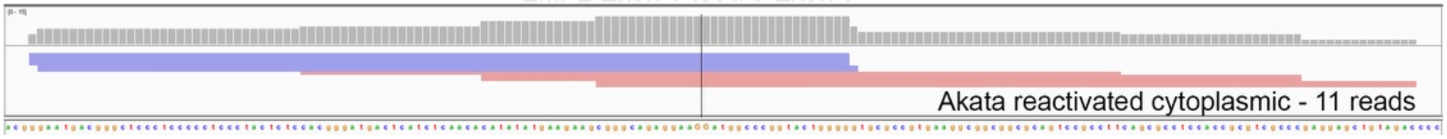

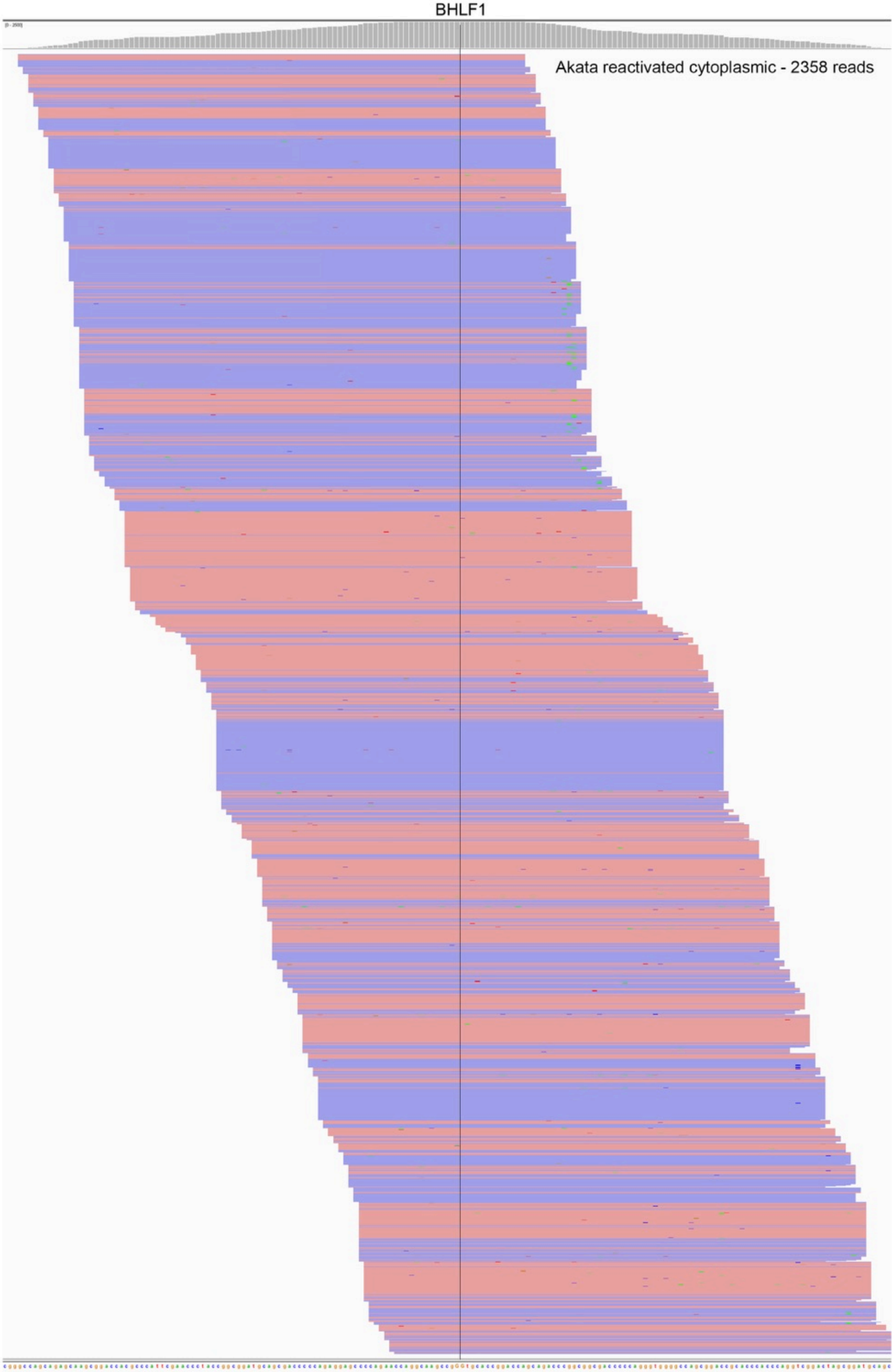
BHLF1
Akata reactivated cytoplasmic - 2358 reads

BGLF2 to BHLF1

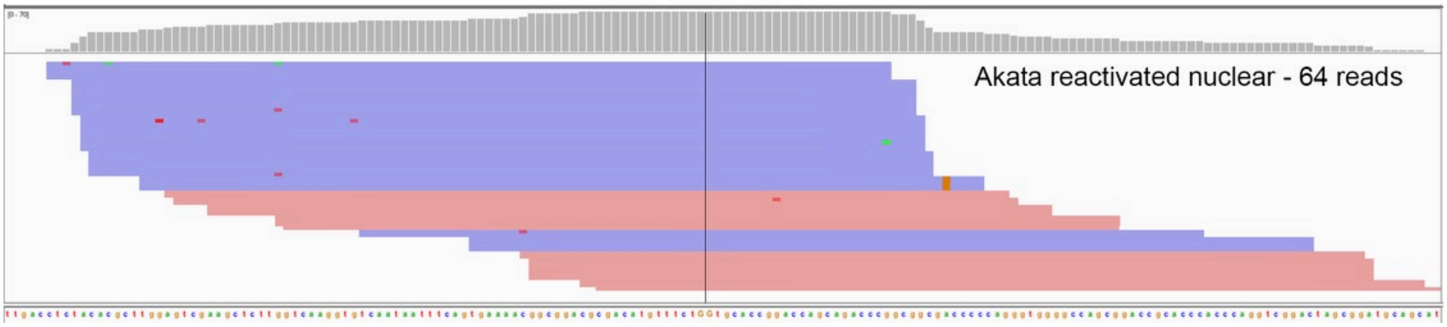

BDLF1 to BHLF1

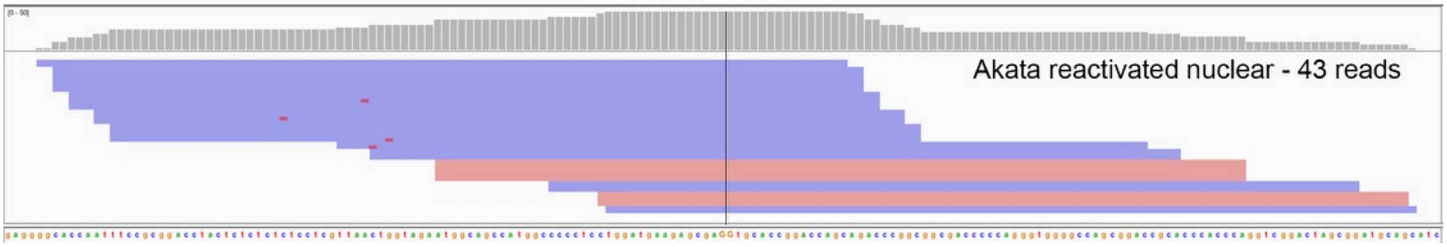

BXLF1 to BHLF1

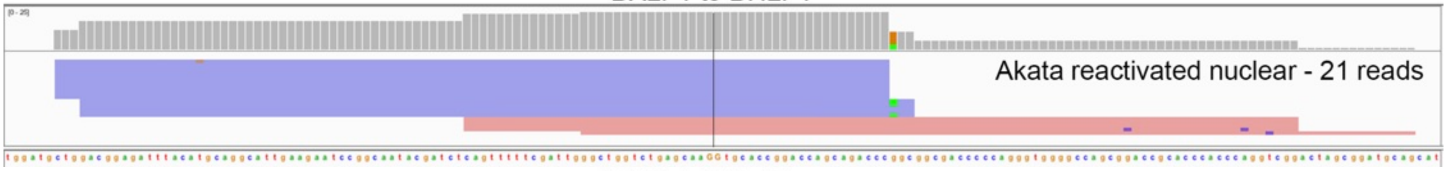

BILF2 to BHLF1

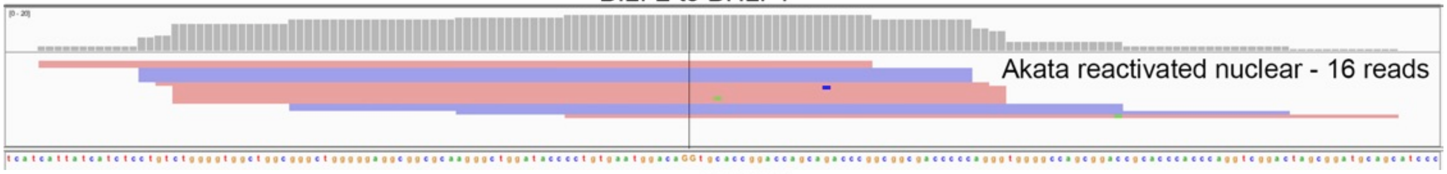

BHRF1

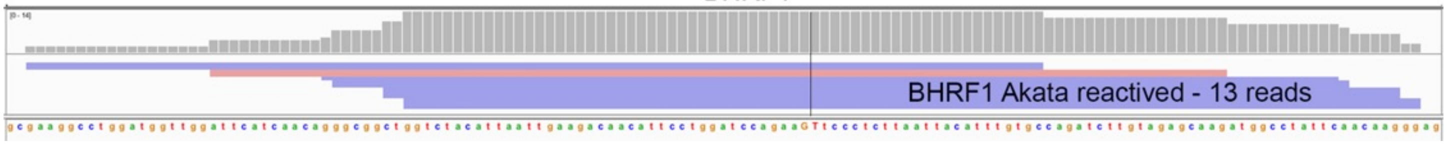

Supplement: S1 File — Reads from each RNase R-seq dataset were aligned to conjoined backsplice junctions using STAR and displayed (squished) on the Integrative Genome Viewer (IGV). Shown for each junction are the dataset with the maximal junction spanning read counts. (PDF) [file ppat.1007206.s011.pdf]
